# Insights into the thermal ecology, physiology, and behavior of a threatened ectothermic specialist from a warming and drying ecoregion

**DOI:** 10.1242/bio.062264

**Published:** 2025-12-23

**Authors:** Brian R. Blais, Maria Vittoria Mazzamuto, John L. Koprowski

**Affiliations:** ^1^ School of Natural Resources and the Environment, University of Arizona, ENR2,1064 E. Lowell St., Tucson, AZ 85721, USA; ^2^ Haub School of Environment & Natural Resources, University of Wyoming, 201 Bim Kendall House, 804 E Fremont St., Laramie, WY 82072, USA; ^3^ Department of Life Sciences and Systems Biology, University of Turin, 10123 Turin, Italy

**Keywords:** Body temperature, Conservation, Endangered species, Infrared thermography, Reptiles, Zoos

## Abstract

Increased heat and drought from Anthropogenic climate change will challenge the adaptive capacity of species, underscoring the need to understand thermal ecology – how organisms behaviorally and physiologically respond to temperature. We used noninvasive infrared thermography (IRT) to examine the thermal ecology of threatened narrow-headed gartersnakes (*Thamnophis rufipunctatus*) in a conservation breeding program at the Arizona Center of Nature Conservation/Phoenix Zoo. From 718 microhabitat and 124 individual measurements, hierarchical models identified extrinsic and intrinsic factors influencing microhabitat usage, body temperature (T_b_), and behavior. Gartersnakes exhibited regional heterothermy, with tails cooler than head and trunk segments. The T_b_ of *T. rufipunctatus* was shaped by perch temperature, perch-air temperature, and whether snakes were visibly exposed or hidden. We documented microhabitat aggregations (≥2 gartersnakes) in ca. 40% of observations, which was best predicted by T_b_. *Thamnophis rufipunctatus* appeared to favor cavity-bearing microhabitats, consistent with wild populations. This first application of IRT to snakes in semi-natural environments, and for *T. rufipunctatus* specifically, provides novel insights to guide more effective field surveillance and conservation management, while demonstrating the broader value of IRT and collaborative *ex situ* studies for wildlife conservation.

## Teaser

**Summary:** Infrared thermography revealed air and surface temperature and behavior to influence body temperature of narrow-headed gartersnakes, providing insights to improve conservation management and monitoring of this threatened species.

## INTRODUCTION

Contemporary rates of rising temperature and prolonged drought under Anthropogenic climate change will challenge the pace at which species can adaptively respond (Bennett et al., 2021; Hof et al., 2011; Mitchell et al., 2018; Radchuk et al., 2019; Visser, 2008), which presents a major threat to Earth's biodiversity (Bellard et al., 2012; Pacifici et al., 2015; Pecl et al., 2017). The impacts of rapid onset changes on bio-available water, metabolism, and reproduction in amphibians and reptiles, for example, can be severe or irreversible (Bickford et al. 2010, Davis et al. 2015, Mitchell et al. 2016). The thermal tolerance and responses to environmental stimuli of a species first require an understanding of its thermal ecology – an organism's behavioral and physiological response to temperature (Bogert, 1959; Camacho et al., 2018; Refsnider et al., 2019). Endothermic animals, such as birds and mammals, generate their own heat through internal metabolism and need mechanisms to conserve it; to maintain body temperature (T_b_), heat produced in the core is distributed to the periphery (Hill et al., 2016; Scholander et al., 1950). Ectothermic reptiles rely on an interplay of environmental stimuli (e.g. conduction, convection, radiation), metabolic physiology, morphology, and behavior to thermoregulate T_b_ to optimal levels to perform natural activities (Christian et al., 2016; Cowles and Bogert, 1944; Cox et al., 2022; Goulet et al., 2017; Kearney et al., 2009).

Many reptiles select microhabitats (e.g. burrows/refuges, basking/perch structures) to thermoregulate as well as perform regular natural behaviors such as foraging, escaping predation, gestating, and aestivation/overwintering among others (Dubiner et al., 2024; Goulet et al., 2017; Harvey and Weatherhead, 2010; Sannolo et al., 2019; Scheffers et al., 2014). Ambient conditions alone may not capture thermal heterogeneity within and among the microhabitat landscape; i.e. microclimate (Campobello et al., 2017; Kearney et al., 2009; Scheffers et al., 2016; Woods et al., 2015). Infrared thermography (IRT; Tattersall, 2016; McCafferty et al., 2021) tools can noninvasively and simultaneously capture the thermal properties of focal species and the microhabitat surfaces being occupied, which can help infer site selection (Blais et al., 2023a; Goller et al., 2014; Krochmal and Bakken, 2003; Signore et al., 2020; Taylor et al., 2021). Ecological insights garnered from IRT include various anatomical, physiological, and behavioral adaptations to thermal environments (Mazzamuto et al., 2023; McCafferty et al., 2021; Mitchell and Clarke, 2019; Monge et al., 2025). Understanding environmental influences of favored microhabitats can guide more effective conservation actions, such as species reintroduction site planning or habitat restoration initiatives (Huey et al., 1989; Luna and Font, 2013).

Assessing thermal physiology in wild systems allow for natural behaviors but complexities and stressors exist (e.g. nutrient requirements, threat of predation), whereas (simple) *ex situ* arenas offer stronger experimental control but could influence behavior or other aspects of thermal ecology, including diel/seasonal changes, sex, gravidity, and age-class, especially for at-risk species (Tattersall, 2016; Taylor et al., 2021). *Ex situ* (e.g. zoos, aquariums) managed semi-natural environments and enrichment strategies that facilitate innate behavior and needs of focal species may provide a compromise through informational feedback (Blais et al., 2023b; Burghardt, 2013; Chiszar et al., 1993; Reading et al., 2013). Zoo populations can present opportunities to closely monitor and gain insight into many natural parameters (e.g. behavior, life history traits, physiology) of a focal species that may otherwise be data deficient or onerous to track *in situ* (Blais et al., 2023b; Minteer et al., 2018; Pritchard et al., 2011; Spooner et al., 2023).

The narrow-headed gartersnake, *Thamnophis rufipunctatus* (Cope, in Yarrow, 1875), is a piscivorous specialist endemic to montane, perennial, cool-water riparian systems (700–2430 m asl) in the Gila River Watershed of central Arizona and western New Mexico (Holycross et al., 2020; Rossman et al., 1996; Wood et al., 2018). Limited information exists on the thermal physiology of *T. rufipunctatus* (USFWS, 2014). Body temperature has been shown to strongly relate to air and surface temperature (Fleharty, 1967) but also water temperature (Hibbitts et al., 2009). This species is active in a wide range of air temperature (T_a_, 11–32°C) and water temperature (T_w_, 12–22°C; USFWS, 2014); active *T. rufipunctatus* can operate at cooler conditions than several congenerics (Rosen, 1991), including sympatric species (Fleharty, 1967). Range-wide population declines (Holycross et al., 2020; Wood et al., 2025a) culminated in the species being listed as threatened under the Endangered Species Act (USFWS, 2014; Wood et al., 2018). Projected loss of suitable environment across time in a warming and drying region presents additional challenges the species is likely to face (Blais and Koprowski, 2024). In response, the Arizona Center for Nature Conservation/Phoenix Zoo (hereafter ACNC) has managed an *ex situ* conservation breeding program for *T. rufipunctatus* since 2006 (Allard and Wells, 2018). The *ex situ* colony at ACNC has provided opportunities to study and learn about this enigmatic species, including insights into behavior and reproductive biology (Blais et al., 2023b; Wood et al., 2025b).

Herein, we conducted a noninvasive IRT study on the ACNC colony to better understand the thermal ecology and physiology of *T. rufipunctatus*. Our objectives were to: 1) model environmental and temporal factors in snake-used versus available microhabitats in two complex naturalistic microcosms; 2) derive T_b_ and thermal physiology metrics; 3) analyze how behavior and environment influence T_b_; and 4) explore relationships of intrinsic and extrinsic factors that may influence aggregation behavior. We expected gartersnakes to select microhabitat types similar to those used in the wild (e.g. refuges with cavities; Holycross et al., 2020; Rosen, 1991) and in relation to thermoregulatory needs. We expected T_b_ measures to be near or slightly above measurements recorded in the wild due to the controlled environment maintained in the *ex situ* location. We use *ex situ* ecological and physiological data to guide conservation of an imperiled ectotherm and inform broader thermal ecology research. Our methods incentivize IRT use in naturalistic settings and strengthen feedback loops and collaboration across the *ex situ*–*in situ* spectrum. Understanding how body temperature, behavior, and microclimate interact can reveal thermal preferences and adaptive challenges terrestrial ectotherms face in a warming, drying climate.

## RESULTS

### General results and temporal conditions

We completed 36 total surveys (1–2 per enclosure per sampling date) between 24 May and 27 September 2019 (sampling date interval: 14±0.7 d); we ceased the project in conjunction with planned maintenance by ACNC in preparation for the overwinter brumation period. Morning shift surveys occurred at 10:12 h on average and afternoon shift surveys at 13:04 h on average (shift interval: 178.1±35.2 min); we omitted two afternoon surveys due to logistical restraints/husbandry priorities. The average survey duration was 13.9 (±5.8) min. Ambient conditions varied temporally between morning and afternoon survey shifts for P_b_ (Wilcoxon test: *P*<0.001; mean change=−2.2 mb), rH (*P*<0.001; mean change=−2.9%), T_a_ (*P*<0.001; mean change=2.7°C), and T_w_ (*P*<0.001; mean change=2.1°C; Fig. S2A,D). Ambient conditions also varied by month (Kruskal–Wallis: all *P*<0.001).

### Microhabitat selection by narrow-headed gartersnakes

We generated a dataset of *n*=718 microhabitat assessments. The thermal heterogeneity of microhabitats varied within and among types (T_s_: 29.8±4.55°C, T_s_-T_a_: 0.4±3.35°C; Table S1, Fig. S3A,B). Although we knew gartersnake total *N* per enclosure survey, we did not always observe all present individuals (detection rate: 60.1±29.7%) and made no attempt to search beyond surface visuals (i.e. beneath substrates, leafy plants, or underwater). Gartersnakes occupied microhabitat types as follows: subterranean (62.9%), cover (22.4%), water (10.3%), ground (2.3%), and plants (2.0%). Twenty-seven of 42 unique microhabitats remained unoccupied during study observation.

Four models were within two corrected Akaike Information Criterion (AICc) units to explain microhabitat selection (Table S2). Only microhabitat type was identified as an important variable at the ≥0.80 threshold score and was the only factor in the top microhabitat usage model (AICc=312.6, weight=0.188, *R*^2^=0.71). After investigating the internal-only microhabitat dataset, there were seven models within two AICc units (Table S2). Again, type was important at ≥0.80 threshold although T_a_ and (internal) T_s_ had scores 0.40–0.60. The model that included those three terms yielded some trends (0.05<*P*<0.10) that *T. rufipunctatus* were more likely to use microhabitats with internal structures when ambient T_a_ increased and microhabitat internal T_s_ decreased. That is, cover microhabitats provided refuge to cool down.

### Thermal ecology of *T. rufipunctatus*

**Fig. 1.**
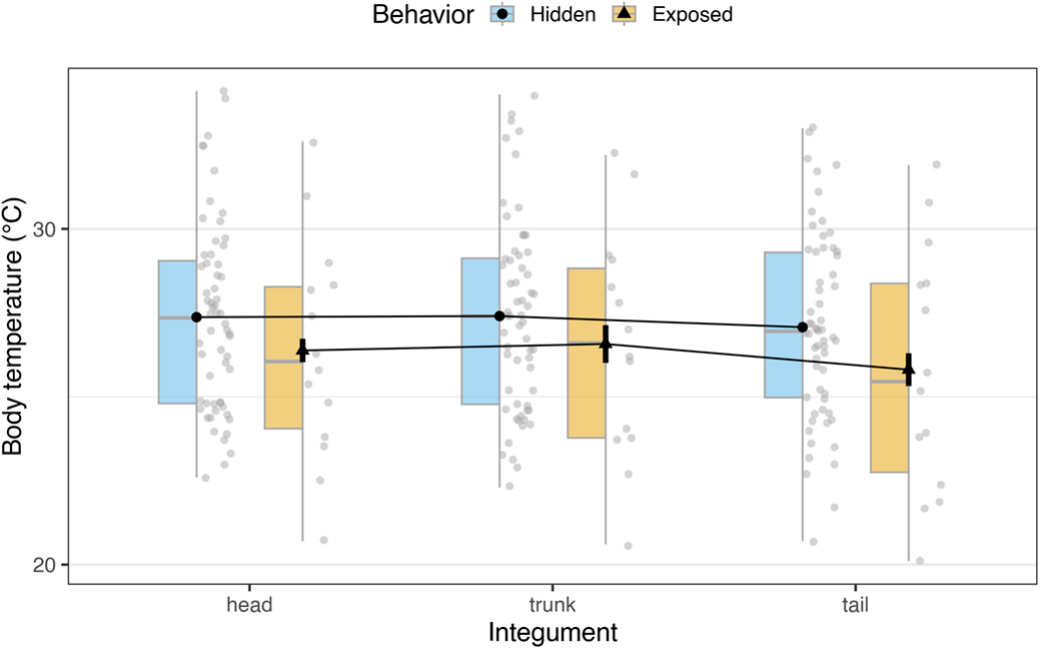
**Regional heterothermy by integument for *ex situ* colony of *T. rufipunctatus* at the Phoenix Zoo, 2019.** Data are external body temperature values (*n*=74) obtained from infrared thermography for gartersnake head, trunk, and tail (post-cloaca) integuments. Data are partitioned by gartersnake behavior, whether they were observably visible and exposed to elements (e.g. basking) or hidden within refuges (e.g. cooling).

We amassed *n*=124 assessments for *T. rufipunctatus*. The body temperature dataset included *n*=117 records after omitting seven events that lacked T_b_ (e.g. indiscernible clustered individuals; snake fled before measurement). From *n*=74 measurement events that had data for all three integuments, there was evidence of regional heterothermy (*F*_144_=14.2, η^2^=0.004, *P*<0.001). Gartersnake head (27.2±2.9°C) and trunk segments (27.2±3.0°C) were equivalent, but tail segments were slightly but significantly cooler (26.8±3.0°C; Fig. 1). Effects from exposure behavior (*P*=0.237), i.e. whether snakes were hidden within refuges (e.g. thermoregulatory cooling) or visibly exposed (e.g. surface basking), or interaction effects (*P*=0.142) were not found. We note that assessing individual integuments on analyses hereafter did not alter results.

**Fig. 2.**
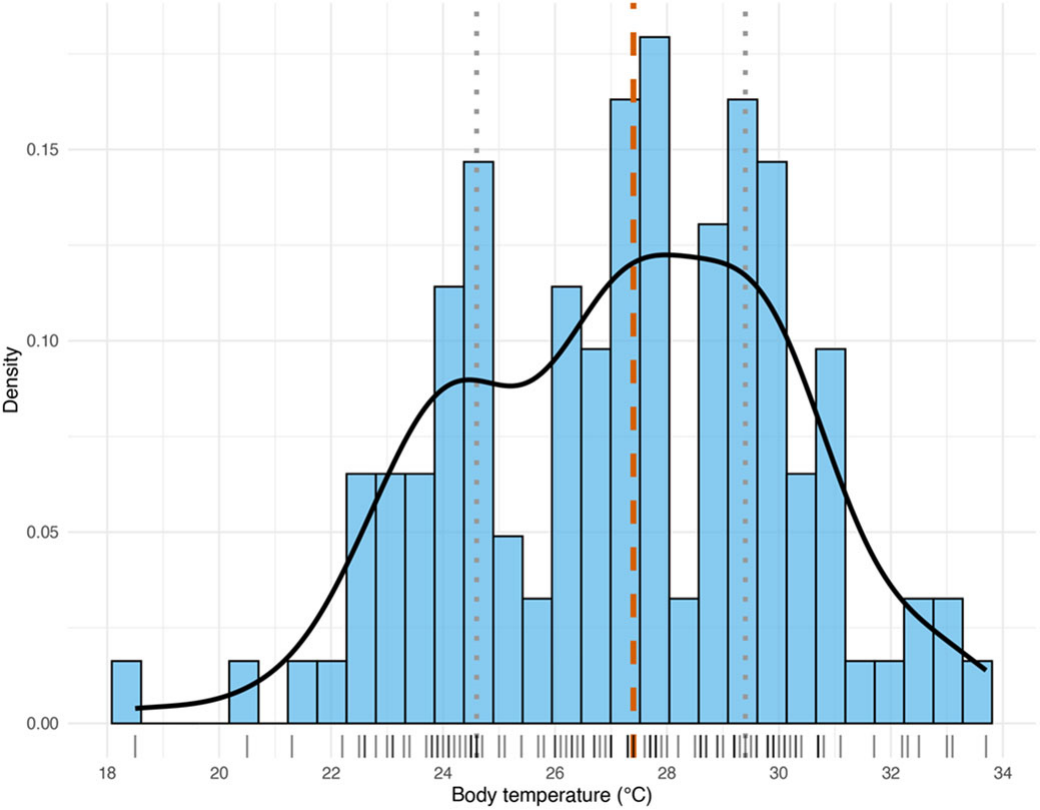
**Distribution of body temperature (*n*=117) for *ex situ* colony of *T. rufipunctatus* at the Phoenix Zoo, 2019.** Vertical lines represent median (dashed, orange) and upper and lower quartile bounds of the preferred body temperature (T_set_; dotted, grey). Tick marks along the x-axis represent individual datapoints.

The population T_b_ metrics for *T. rufipunctatus* (*n*=117) were 27.2±2.94°C. This central tendency and dispersion varied from populations in older studies (*Z*>3, *P*<0.001; Fleharty, 1967; Rosen, 1991; Hibbitts et al., 2009) but was comparable to a more recent assessment (*Z*=1.7, *P*=0.082; see Jennings and Christman in USFWS, 2014; Table 1). The preferred body temperature was as follows: median (i.e. T_set_)=27.4°C, T_set_ lower bounds=24.6°C, T_set_ upper bounds=29.4°C, T_set-range_=4.8°C (Fig. 2). Thermoregulatory accuracy (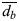) was 0.2±2.94°C (range: −6.3 to 8.9°C; Fig. S4A) and absolute value (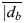
|) was 2.4±1.71°C (range: 0–8.9°C; Fig. S4B); the minimum value was for an individual in water and the maximum value was for an individual undergoing ecdysis. Daily maximum T_b_ (VT_max_) across surveys was 30.1±2.67°C. Notable remarks for VT_max_ are that max temperature occurred during afternoon scans for eight of ten survey days, for hidden (i.e. unexposed) individuals seven of ten times, and twice for individuals undergoing ecdysis.

**Table 1.** Comparative body temperatures (°C) of *T. rufipunctatus* during its active season (ca. March–November)

*n*	Mean (°C)	s.d. (°C)	Min.–max. (°C)	Method	Source
31	24.7	±2.2*	20.0–29.0	QCT	Fleharty, 1967
18	25.0	±3.7	19.2–31.4	QCT	Rosen, 1991
60	26.4	±2.7	19.8–30.4	QCT	Hibbitts et al., 2009 ^§^
15	26.5	±4.3	17.3–32.0	QCT	Jennings and Christman^¶^
8	NA	NA	10< T_b_ <35^‡^	TRT	Nowak^¶^
117	27.2	±3.0	18.5–33.7	IRT	Present study

Methods of assessment include cloacal temperature (QCT; quick-read thermometer), temperature-sensing radio transmitter (TRT; internally implanted), and infrared thermography (IRT; external/dermal). Sample size (*n*) may include repeated measurements across time. *=converted from standard error; ^‡^=inferred from graphical data (active season only); ^§^= author provided additional data; ^¶^=unpublished report, see Holycross et al. (2020); USFWS (2014).

**Fig. 3.**
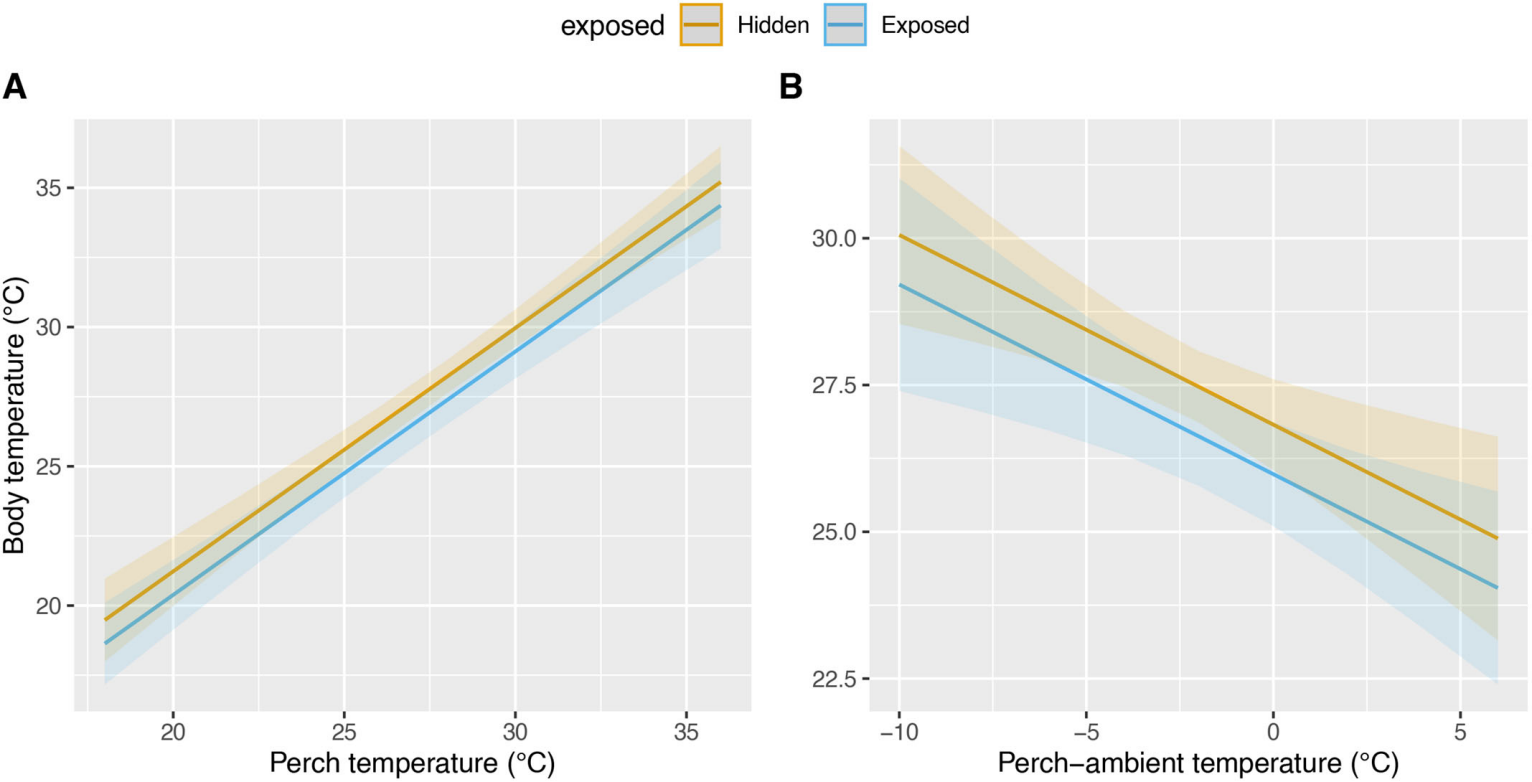
**Predicted change in body temperature for *ex situ* colony of *T. rufipunctatus* at the Phoenix Zoo, 2019.** (A) Perch surface temperature and (B) the difference in perch temperature from ambient. Data are partitioned whether individuals were visibly exposed or hidden at a microhabitat.

Four competing LMM models (<2 ΔAICc) best described T_b_ of *T. rufipunctatus* (Table S3). The best performing model included T_perch_, T_s_-T_a_, exposed, and shared microhabitat behavior (AICc=391.3, weight=0.184, *R*^2^=0.83); only the latter term failed to meet the importance threshold (>0.8). Competing models with additional terms had relative importance well below the 0.8 threshold and their inclusion failed to improve AICc >2. For each unit increase in T_perch_ or T_s_-T_a_ in the optimal model, T_b_ increased by 0.87°C [confidence intervals (c.i.): 0.74–1.01°C; Fig. 3A] and decreased by 0.32°C (c.i.: 0.14–0.51°C; Fig. 3B), respectively, after accounting for other terms. A categorical shift from hidden to exposed behavior (i.e. surface-visible) yielded a 0.42°C decline in T_b_ (c.i.: 0.08–0.76°C), holding other terms constant.

### Behavioral assessment

**Fig. 4.**
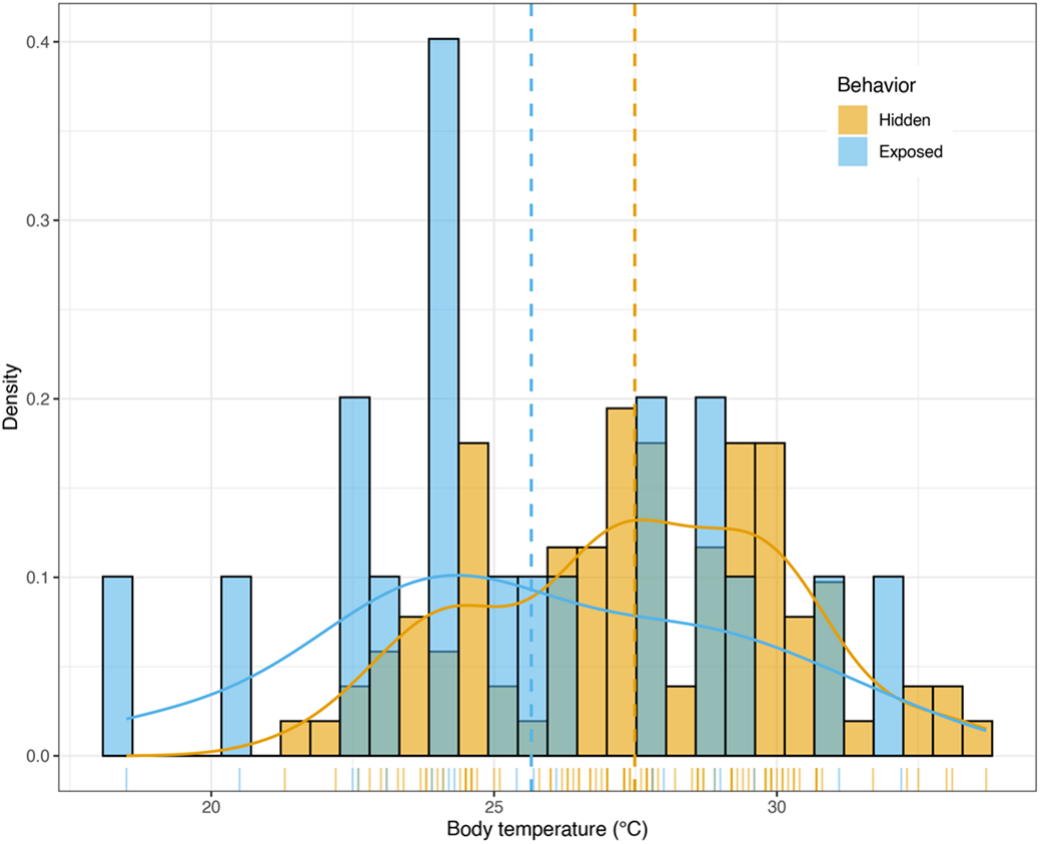
**Behavioral differences in external body temperature (*n*=117) for *ex situ* colony of *T. rufipunctatus* at the Phoenix Zoo, 2019.** Data are partitioned by visually exposed (blue) versus hidden (orange) gartersnakes. Vertical dashed lines indicate sample means and tick marks along the x-axis represent individual datapoints.

We observed actively moving individuals only five times (4.0%; Table 2). We observed only four instances of activity in water but note that the complexity of the aquatic environment and natural behavior of the species could have obscured detection. Gartersnakes were partially or entirely exposed – to the visual scan of the observer – in 21 of 124 detections (16.9%). We encountered more exposed gartersnakes during morning surveys (23.7%) than afternoon surveys (6.3%, χ^2^=5.2, *P*=0.023; Table 2). Hidden snakes were warmer (27.5±2.72°C) than exposed individuals (25.7±3.60°C, *P*=0.045; Fig. 4), suggestive of afternoon retreat into refuges once upper bounds of preferred temperature were obtained from morning warm up. Gartersnakes were warmer in the afternoons (29.4±2.18°C) than mornings (25.8±2.51°C, *P*<0.001) but there was no interactive effect of exposure (*P*=0.123).

**Table 2.** Frequency of behavior events by diel period for *ex situ* colony of *T. rufipunctatus* at the Phoenix Zoo, 2019

Disposition	Morning		Afternoon		Cumulative	
	Freq. (%)	Mean T_b_ (±s.d.)	Freq. (%)	Mean T_b_ (±s.d.)	Freq. (%)	Mean T_b_ (±s.d.)
Active	1 (1.4)	28.0 (--)	2 (4.4)	30.0 (3.1)	3 (2.6)	29.3 (2.5)
Inactive	71 (98.6)	25.8 (2.5)	43 (65.6)	29.3 (2.2)	114 (97.4)	27.1 (2.9)
Exposed	16 (22.2)	24.8 (3.1)	3 (6.7)	30.4 (2.3)	19 (16.2)	25.7 (3.6)
Hidden	56 (77.8)	26.1 (2.3)	42 (93.3)	29.3 (2.2)	98 (83.8)	27.5 (2.7)
Solitary	24 (33.3)	26.4 (2.6)	19 (43.2)	29.5 (2.4)	43 (37.1)	27.7 (2.9)
Aggregated	48 (66.7)	25.6 (2.4)	25 (56.8)	29.3 (2.1)	73 (62.9)	26.8 (2.9)

Disposition refers to gartersnake behaviors, recorded *ad libitum* during surveys: Active=actively moving or swimming; Inactive=immobile/stationary; Exposed=partially or fully surface-visible to observer; Hidden=unexposed; Solitary=1 gartersnake per microhabitat; Aggregated=≥2 gartersnake per microhabitat. Freq.=frequency of events (%); *T_b_*=body temperature.

Exposed behavior was best explained by T_a_ and T_s_ ex-in (AICc=46.9, weight=0.461, *R*^2^=0.78; Table S4). For each unit increase in T_a_, the odds of shifting between hidden to exposed behavior decreased by 0.33 times (c.i.: 0.11–0.92), i.e. −67%; gartersnakes were most likely to be hidden when late-morning, early-afternoon air temperature reached ca. 28°C (Fig. S5). There were some trends that probability of exposure increased with greater differences between external and internal microhabitat surface temperature but with uncertainty (confidence intervals crossed 1).

Gartersnake aggregations were observed 31 times (39.7% of occurrences). The mean number of aggregated snakes was 2.5 (±0.77). The maximum number of snakes sharing a given microhabitat was four, which was recorded five times; most aggregations occurred within refuges such as small hide boxes or hibernacula. Aggregations occurred more often during the morning (64.5%) than the afternoon (35.5%; Table 2), and aggregations per month from May through September (2019) were three, six, four, nine, and nine, respectively. There were no significant differences in T_b_ for gartersnakes in aggregations versus solitary ones (*F*_114_=2.6, *P*=0.111).

**Fig. 5.**
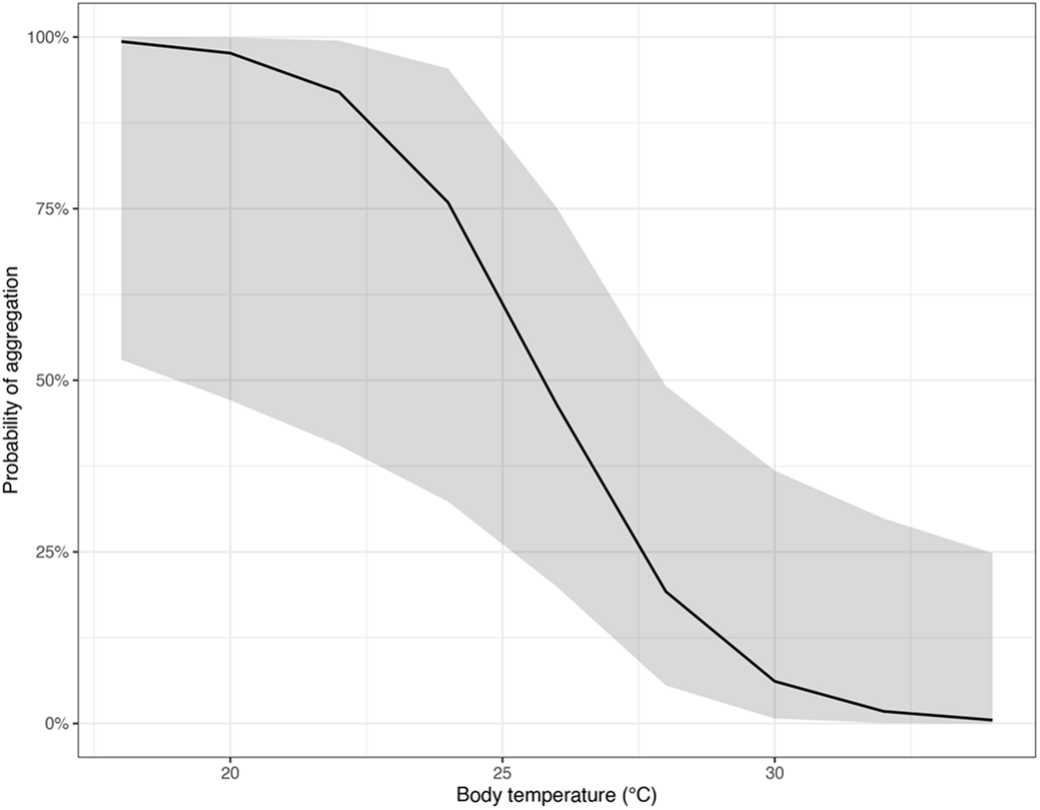
**Probability of aggregation behavior based on body temperature for *ex situ* colony of *T. rufipunctatus* at the Phoenix Zoo, 2019.** Aggregation is defined as ≥2 snakes (versus solitary individuals) at a given microhabitat.

Four competing GLMMs best explained aggregation behavior at microhabitats (Table S5). All contained average T_b_ from present individuals, which was the only factor meeting the importance threshold. The top performing model included average T_b_ and T_perch_ (AICc=96.1, weight=0.200, *R*^2^=0.48). After accounting for other terms, each unit increase in average T_b_ decreased the probability of aggregation by 0.52 times (c.i.: 0.30–0.91), i.e. 48% decline: warmer gartersnakes were less likely to aggregate (Fig. 5). There were trends that increasing T_perch_ led to higher aggregation probability but with uncertainty (confidence intervals crossed 1). Three competing models best explained aggregation quantity, each contained average T_b_ from present individuals and/or T_perch_ (Table S5). Neither of these factors, however, met the variable importance threshold (<0.8) and did not appear to influence aggregations due to uncertainty about the confidence intervals (crossing 1). That is, the tested variables failed to explain an influence of aggregation quantity in the *T. rufipunctatus* colony.

## DISCUSSION

We used IRT, a reliable and noninvasive method, to infer surface body temperature for a colony of threatened *T. rufipunctatus* in naturalistic *ex situ* mesocosms. We examined extrinsic and intrinsic factors that influence microhabitat usage, T_b_, and behavior. These insights into the seldom assessed thermal ecology and physiology of *T. rufipunctatus* serve as informational feedback loops to increase knowledge and steer conservation strategies across the *ex situ*–*in situ* spectrum. To our knowledge, this is the first application of IRT for *T. rufipunctatus* and on snakes in enriched mesocosms.

### Unraveling the thermal ecology of a threatened, limited-range ectotherm

Ecological insight into *T. rufipunctatus* ecology and life history is warranted (USFWS, 2014), and opportunities remain to investigate the physiology and thermal ecology for the species. The range of body temperatures of *T. rufipunctatus* from this study was slightly warmer than reported from some wild populations (Fleharty, 1967; Hibbitts et al., 2009; Rosen, 1991). Snakes can maintain T_b_ higher in lab settings than wild (Cox et al., 2023). However, the T_b_ exhibited were equivalent to the most recent wild assessment (see Jennings and Christman in USFWS, 2014), which may better reflect contemporary climate shifts across the species' range (Blais and Koprowski, 2024; Pilliod et al., 2024). The ACNC colony's T_b_ and T_set_ were also within the range of a relatively high stable phase activity temperature exhibited by *T. rufipunctatus* and syntopic congenerics (Rosen, 1991). Several factors were influential in predicting T_b_. The most prominent were perch temperature, the difference in microhabitat surface temperature from ambient, and behavior (particularly, exposure). As perch temperature rose, so did T_b_ – concordant with findings in wild populations (Fleharty, 1967; USFWS, 2014) and other regional herpetofauna (Blais et al., 2023a; Dubiner et al., 2024). Body temperature also increased as ambient air temperature increasingly exceeded microhabitat surface temperature (T_s_-T_a_; see Fig. 3B). These emphasize heterogeneity of the mesocosm environment and allowed for behavioral shifts among microhabitats to occur.

Relatively few snakes have been documented to exhibit regional heterothermy (Cox et al., 2023, 2024). Our findings add *T. rufipunctatus* to this list with warmer head/trunk than tail patterns, similar to other temperate congenerics (Gregory, 1990). These differences were significant at about 0.4°C, which is less variable than observed in other species (e.g. 1.7–2.8°C in *Diadophis punctatus*; Cox et al., 2024). This may be due to adaptations as a semi-aquatic piscivore operating at cooler temperatures than congenerics (Fleharty, 1967; Rosen, 1991), and a prehensile tail important for underwater foraging strategies (e.g. anchoring in swift-currents; Holycross et al., 2020). Diel/seasonal timing, instrument type, and internal-specific measurement could also affect heterothermy inference. Because heterothermy can persist across behaviors (e.g. exposed versus hidden) and regardless of thermal environments – albeit at lesser extents as temperature increases, endogenous mechanisms may be at play in thermoregulating vital areas such as the head/brain (Cox et al., 2024; Tattersall et al., 2006). Regional heterothermy in *T. rufipunctatus* may have more to do with physiological necessities than environmental influence; further research on underlying functional mechanisms of regional heterothermy is warranted.

Exposed behavior also influenced T_b_ but, interestingly, hidden snakes tended to be warmer than exposed snakes. This result is likely temporal. More frequent exposed individuals in the morning hours (cooler body temperatures, basking) versus warmer but more likely hidden gartersnakes in the afternoon could be explained by afternoon retreats into refuges when mean T_b_ approximated the T_set_ upper bounds; gartersnakes likely reached their upper preferentia sometime in the mid to late morning and had retreated into cover by time the early afternoon surveys occurred. This finding is supported by our assessment of exposed behavior, which was influenced by ambient air temperature; gartersnakes were more likely to be hidden once ambient temperature exceeded ca. 28°C across the survey season. These findings lend further support of a morning warmup and afternoon retreat in *T. rufipunctatus* (see literature in USFWS, 2014) and other snakes (Venegas-Barrera et al., 2025). Because visual observations were done *ad libitum*, we acknowledge uncertainty whether individuals had recently shifted from basking to cooling or vice versa.

Numerous factors can influence microhabitat selection by reptiles, including thermal and non-thermal properties (Blais et al., 2023a; Campobello et al., 2017; Mushinsky and McCoy, 2016; Ortega et al., 2019). *Thamnophis rufipunctatus* is often detected atop or within riparian rock piles, vegetation, and other structures with areas for quick escape (Hibbitts et al., 2009; Holycross et al., 2020; USFWS, 2014); they have also been observed occupying pipes and other manmade structures in parts of its range (see Nowak in USFWS, 2014). For the *ex situ* colony of *T. rufipunctatus* at the ACNC, we found that microhabitat type was the best predictor of selection, particularly cover objects that had internal cavities for refuge. Despite microclimate heterogeneity among the microcosms, no other factor appeared to influence microhabitat occupancy in this study; untested variables (e.g. dimensionality, solar radiation) may provide further resolution (Ortega et al., 2019).

Squamate reptiles, including *Thamnophis* gartersnakes, aggregate for a variety of reasons across inter/intraseasonal periods, such as overwinter denning, beneath cover during active seasons, gestation, and for mating (Aleksiuk, 1977; Gardner et al., 2016; Graves and Duvall, 1995; Gregory, 2004). Vomeronasal mechanisms guide individuals into aggregations (Aubret and Shine, 2009; Heller and Halpern, 1982). Aggregations in wild *T. rufipunctatus* populations are sparsely reported (Blais and Lashway, 2018; Holycross et al., 2020; USFWS, 2014). Our study is the first to examine links between thermal ecology and aggregation behavior in *T. rufipunctatus*. We identified a relationship between T_b_ and whether individuals aggregated or not; warmer gartersnakes were less likely to (or continue to) aggregate. None of our tested variables, however, explained aggregation cohort size. Congenerics in temperate zones are known to aggregate to prevent water loss, albeit concentrated during overwinter denning (Costanzo, 1989), whereas during the active season, aggregations to reduce cooling rates tend to favor neonates/younger (i.e. smaller) snakes (see Aubret and Shine, 2009 for a review on squamate aggregation). A ‘selfish herd’ defense mechanisms for aggregation, especially during vulnerable periods such as female gestation (Gregory, 2016) could also exist; one female reproduced during our study year (Wood et al., 2025b). More recently, some *Thamnophis* were found to aggregate socially by engaging in nonrandom social interactions (Skinner and Miller, 2020). Failing to find statistical support among our tested variables for aggregation cohort size lends to two basal explanations: 1) the available data were insufficient to test the variables and/or, 2) we failed to examine the variable(s) that explain clustering, such as social reasons (Skinner and Miller, 2020). Our noninvasive ‘no touch’ design prohibited closer examination for sex and individual ID, though we recommend that future investigations apply mark-recapture techniques (e.g. PIT tags, nontoxic paint markings) to track any social, reproductive, or rate-of-change evidence therein. In the wild, observers should take note of microhabitat type/structure, sex, size, gravidity, and developmental age class of aggregating individuals.

### Implications across the *ex situ*–*in situ* spectrum

There were several indicators that the *T. rufipunctatus* in the ACNC colony are exhibiting behavioral and physiological traits akin to those observed in wild populations. Body temperatures were equivalent to the most recent known assessment in the wild (see Jennings and Christman in USFWS, 2014), and thermoregulatory accuracy was comparable to typical values in other squamates (Hertz et al., 1993; Ivey et al., 2020). Moreover, the lack of month significantly influencing T_b_ infers that *T. rufipunctatus* were able to thermoregulate across the active season (Ivey et al., 2020). Taken together, this suggests there was sufficient microclimate heterogeneity for the colony to adequately thermoregulate; individuals on the outer margins occurred during spring mornings or in water (cooler end) or were undergoing ecdysis in summer afternoons (warmer end). Behaviorally, gartersnakes were seldom observed moving and usually were well hidden and sometimes undetectable to the brief visual scan despite the finite environment. Similar traits have been observed in the wild (Holycross et al., 2020; Rosen, 1991; USFWS, 2014). Enriched *ex situ* populations managed in semi-natural environments can exhibit innate behaviors and physiology (Blais et al., 2022; Burghardt, 2013; Rabier et al., 2022), which may be key for producing fit individuals to maximize opportunity for *in situ* conservation success (Blais et al., 2025; Choquette et al., 2023; Gross et al., 2024; Péchy et al., 2015; Roe et al., 2015). Collectively, these findings support that the *ex situ* colony is engaging in direct measures of natural defense and survival behaviors (Chiszar et al., 1993) while helping build the ecological knowledge base for *T. rufipunctatus* (Blais and Lashway, 2018; Holycross et al., 2020).

When *ex situ* populations exhibit normal traits and processes, the inferences obtained from empirical research may better reflect conditions expected in the wild and guide more informed field sampling and monitoring strategies (Blais et al., 2023b; Chiszar et al., 1993; Roe et al., 2015). Locating the rare and declining *T. rufipunctatus* in the wild can be challenging (Ryan et al., 2019; USFWS, 2014). Deciphering the relationships between environmental temperatures and both body temperature and behavior may hint at when and where to find snakes (Davis et al., 2008). We provide further evidence (Holycross et al., 2020; USFWS, 2014), for example, that surveys earlier in the day (T_a_ <28°C) during the late spring through summer months may be optimal for detecting basking individuals, whereas searches beneath cover may be more fruitful during mid-day heat when individuals have likely retreated to cool off.

### Conservation benefits and future directions

*Thamnophis rufipunctatus* have declined throughout their range – attributed to wildfire, habitat degradation/loss, and competition/predation from non-native invasive species (Holycross et al., 2020; USFWS, 2014) – culminating in small, genetically isolated, and vulnerable populations (Wood et al., 2018, 2025a). Distributions correlate with overwinter temperature and dry season precipitation (Blais and Koprowski, 2024). Concerningly, their occupied region is expected to get warmer and dryer (Archer and Predick, 2008; Cook et al., 2015; Georgescu et al., 2022), with substantial reduction in suitable environmental range projected under future emissions scenarios (Blais and Koprowski, 2024). Conservation recommendations call for further examining ecology and life history, managing habitats for occupancy, and increasing genetic diversity and viability of populations through translocations, including individuals from conservation breeding programs (Holycross et al., 2020; USFWS, 2014; Wood et al., 2025a). The insights from this study mark a step in understanding the thermal ecology of this riparian-dependent, cool-tolerant ectotherm in a range that is likely to be adversely affected by climate change (Blais and Koprowski, 2024).

The critical next steps include assessing thermal maxima and adaptive potential in *T. rufipunctatus* and other ectotherms under threat of climate change (Bodensteiner et al., 2021; Griffis-Kyle et al., 2018; Hof et al., 2011; Kearney et al., 2009; Monge et al., 2025). Advances in IRT technology enable accurate and real-time monitoring of body and environmental temperature across thermal landscapes (Blais et al., 2023a). Paired with remote cameras, IRT may reveal diel, seasonal, and long-term spatiotemporal thermographic trends *in situ* and *ex situ* (Alujević et al., 2025; Mochales-Riaño et al., 2024; Rowe et al., 2020). Multidisciplinary studies integrating IRT and genomics could diagnose evolutionary potential of thermal tolerance traits (Monge et al., 2025). Spatially broader IRT scans may help detect ‘climate refugia’ (Keppel et al., 2015; Scheffers et al., 2014; Suggitt et al., 2018) critical for future management (Blais and Koprowski, 2024; USFWS, 2021). Resolution into other dynamics of behavior and thermal ecology by developmental age class – such as diel/nocturnal activity, brumation hibernacula, and gestation sites – would be informative (Aleksiuk, 1977; Aubret and Shine, 2009; Blais et al., 2023a; Krochmal and Bakken, 2003; Reiserer et al., 2008; Signore et al., 2020).

This study and others (Fleharty, 1967; Rosen, 1991) shows that *T. rufipunctatus* depends on microhabitat structure, indicating the importance of thermal heterogeneity. For management purposes, maintaining and restoring microhabitat structure and complexity across riparian and upland buffer zones is likely paramount (Scheffers et al., 2014; Suggitt et al., 2018; Woods et al., 2015); artificial cover (e.g. concrete boards) may provide substitutes when natural refuges are lost (Lelièvre et al., 2010). Potential collaborations among wildlife managers and zoos/aquariums should promote naturalistic environments in conservation breeding programs to promote essential behaviors (e.g. sociality, reproductive needs, foraging strategies, etc.; Blais et al., 2022; Eisenberg and Kleiman, 1977; Minteer et al., 2018). Doing so may present optimal arenas for *ex situ* studies benefiting *in situ* conservation and translocations (Blais et al., 2025; Gross et al., 2024; Spooner et al., 2023). *Ex situ* managers of *T. rufipunctatus* or other terrestrial ectotherms should monitor responses and maintain key microhabitat conditions, e.g. hides/burrows, water temperature (Hibbitts et al., 2009; Holycross et al., 2020; Murphy et al., 1994; Rosen, 1991). A deeper exploration into intrinsic, extrinsic, and spatiotemporal drivers of thermal ecology, occupancy, and microhabitat selection will refine species and habitat conservation strategies.

## MATERIALS AND METHODS

### System and experimental design

The ACNC's narrow-headed gartersnake enclosures and husbandry management have been adaptively retrofitted to incorporate naturalistic conditions aiming to optimize snake welfare and stimulate reproductive activity (Allard and Wells, 2018). Gartersnakes were managed communally for sociality and pedigree purposes (Blais et al., 2022; Wood et al., 2025b) in two (4.88×2.44×2.44 m) adjacent climate-controlled walled enclosures with a translucent sheet roof that allowed for natural solar photoperiodism. Each enclosure had a dug-out and lined pond with circulated and aerated water and a small waterfall to simulate natural plunge-pool stream habitat. Live fish were managed in the aquatic zones for natural foraging. Each enclosure had a sunken and climate-controlled hibernaculum chamber for brumation; further details of enclosure design, environmental parameters, and cohort origins/pedigree are described elsewhere (Blais et al., 2022, 2023b; Wood et al., 2025b).

We enumerated microhabitats available across both enclosures (*n*=42) and categorized their type as cover (e.g. bark slabs, plastic zoological hide boxes, PVC half-pipes), ground (e.g. single/stacked rock slabs, open areas), plants (e.g. leafy vegetation, branches/sticks), subterranean (i.e. hibernacula and its cover slab), and water (e.g. pool with submerged and partially-emergent river rocks). The program's design aimed to simulate environmental heterogeneity and, because cohabitation and free-movement was unimpeded (i.e. the snakes choose when/where to move, feed, and engage in brumation), innate behaviors are stimulated, including aquatic-adjacent basking, courtship, and reproduction (Blais et al., 2022, 2023b). The enclosures thus act as mesocosms comparative to natural systems used by *T. rufipunctatus*.

We conducted biweekly IRT surveys, May–September 2019, coinciding within the narrow-headed gartersnake active season and especially during the gestation/parturition period (Blais et al., 2023b; Goldberg, 2003; Holycross et al., 2020). We performed separate late morning and early afternoon survey shifts in each enclosure (i.e. four surveys/date) to encompass diel activity trends (Holycross et al., 2020); snakes can exhibit daily thermoregulatory patterns that include a morning ramp up and a mid-day stabilization phase (Venegas-Barrera et al., 2025). At the beginning of each survey, we used a handheld anemometer (Kestrel 5000, Nielsen-Kellerman Co., Boothwyn, Pennsylvania, USA) to measure ambient conditions at 1 m including air temperature (T_a_, ±0.1°C), relative humidity (rH, ±0.1%), and barometric pressure (P_b_, ±0.1 millibars). Water surface temperature (T_w_, ±0.1°C; HI98129, Hanna Instruments, Smithfield, Rhode Island, USA) was recorded daily by ACNC staff. Because certain environmental stimuli may influence gartersnake behavior and body temperature (Rosen, 1991; Row and Blouin-Demers, 2006; Venegas-Barrera et al., 2025), we categorically estimated cloud cover and recent rainfall (scored binomially if occurred ≤48 h); we obtained precipitation data from a nearby weather station (<5 km, Tempe, AZ, USA). Photoperiod and seasonal climate patterns (e.g. summer monsoonal onset) in Phoenix, Arizona, are comparable to natural populations circa ±2° latitude away (Blais et al., 2023b). We preliminarily used Wilcoxon rank sum tests to compare ambient conditions (T_a_, rH, P_b_; nonnormally distributed) and T_w_ between enclosures. Because none of the tested conditions differed between enclosures (Wilcoxon tests: *P*>0.05), we used a combined dataset for analyses.

To derive surface temperature (T_s_) of microhabitats, we used a FLIR E8 infrared thermal camera (FLIR Systems, Wilsonville, OR, USA) to capture multispectral thermograph/photograph combinations [resolution=320×240 (76,800) pixels, accuracy ±2%; thermal sensitivity <0.06°C] at a height approximately 0.5–1 m (Blais et al., 2023a). We ensured emissivity was set to 0.97, commonly used with reptiles (Barroso et al., 2016; Blais et al., 2023a; Luna and Font, 2013). For microhabitats with both external and internal surfaces (e.g. bark slabs, hide boxes), we first scanned the exterior surface before gently lifting the object to scan the underlying substrate surface; this external-internal process may better encompass the microclimate heterogeneity available to snakes (Cox et al., 2023).

When gartersnakes were visibly present on or within microhabitats, we captured additional images that included full body length (dorsal side) whenever possible (see Fig. S1). Dermal surface temperature of relatively small squamates captured by IRT, for example, is correlated with cloacal or internal body temperature (Barroso et al., 2016; Rowe et al., 2020; Tattersall, 2016), which, for snakes, is related to environmental temperature (Cox et al., 2023, 2024). We scored microhabitat occupancy (snake absence, presence) and quantified aggregations (i.e. *n*≥2; Gregory, 2004). To minimize disturbance, only a single observer (B.R.B.) performed surveys, and gartersnakes were not handled due to noninvasive design. Inherently, some individuals became alerted to observer presence, including when refuge covers were temporarily lifted for scans. We assigned binary scores to the following behaviors: ‘exposed’ (i.e. partial or entire body exposed to surface elements and visible to observer upon first detection=1; or hidden entirely within a microhabitat=0); ‘moving’ (if a gartersnake was actively locomoting across terrestrial or aquatic zones=1, immobile=0); and ‘shared’ (if a given microhabitat was shared with more than one individual=1, or not i.e. solitary=0). The number of gartersnakes present in each enclosure was known prior to each survey (colony maximum *N*=11 mature individuals) but not all individuals were detected each survey; we only analyze data on detected snakes. Sexes within enclosures were mixed for pedigree management (Wood et al., 2025b), but individual ID or sex could not be discerned due to our noninvasive design. Body size is not known to influence external body temperature in snakes (Cox et al., 2023; Venegas-Barrera et al., 2025) but can sometimes vary by sex (Rosen, 1991; Venegas-Barrera et al., 2025). This research was conducted under board approval from the Arizona Center for Nature Conservation/Phoenix Zoo's Conservation Department; the ACNC is accredited by the Association of Zoos and Aquariums (AZA) and meets AZA standards of animal care and welfare.

### Thermographic assessment

We used diagnostic tools in FLIR Tools software v. 6.4 (FLIR Systems) to generate descriptive statistics (±0.1°C), where each pixel in a thermograph represented a datapoint. Parallel to Blais et al. (2023a). We used polygon tool functions in attempt to capture the maximum coverage of microhabitat T_s_ without overlapping onto adjacent microhabitats or snake body surfaces. Because surface water temperature measurements were equivalent between IRT and ACNC's water meter (*t*_17_=0.4, *P*=0.69), we retained only the IRT data hereafter for consistency.

We used a combination of spot, line, and polygon functions in FLIR Tools (Blais et al., 2023a) to generate T_b_ (±0.1°C) of gartersnakes. We assessed regional heterothermy (i.e. T_b_ differences by body segments; Sannolo et al., 2014; Barroso et al., 2016, 2020) by investigating dorsal integuments separately as follows: head measurements included the center of the skull and from the snout to back of head when possible; trunk (mid-body) measurements included linear segments (lines, polygons) from behind the head to approximately the cloaca (Cox et al., 2023) – ca. 0.67 of the body length; and tail measurements (when observable) occurred along the posterior third of individuals, where tail width tapers after the cloaca. If an integument had >1 measurement, we estimated averages; we omitted an individual's integument sections if it was not visibly discernable from thermograph/photograph combinations. In preliminary testing, we found no differences between line or polygon functions which suggests either can be used advantageously pending the positioning of subjects. Because this study uses IRT to gain insights into the thermal ecology and physiology of a threatened ectotherm in a naturalistic zoological setting, we were more interested in comparative relationships of body temperature and environment – more applicable for conservation practitioners – rather than finite calibrations of internal T_b_ (Blais et al., 2023a; Mazzamuto et al., 2023; Playà-Montmany and Tattersall, 2021).

### Statistical analyses

Throughout and where applicable, we used Shapiro-Wilk tests and QQ-plots to assess data normality and a Spearman correlation matrix to ensure highly correlated variables (|*r*|>0.7) were not combined in downstream analyses. We used Wilcoxon tests and Kruskal–Wallis test to assess differences in ambient conditions (T_a_, rH, P_b_, T_w_) by diel shift and month, respectively. To examine environmental and temporal influences of microhabitat occupancy (used=1, available=0; Objective 1), we used mixed effect logistic regression (GLMM) from the R package lme4 (Bates et al., 2015) against the following suite of predictors: mean microhabitat surface temperature (T_s_), the difference in mean T_s_ to ambient air temperature (T_s_-T_a_), relative humidity (rH), microhabitat type, diel shift, and month. We set microhabitat ID as a random effect. We used a function in the glmulti package (Calcagno, 2020) to exhaustively automate the top performing models within two corrected AICc units from the global lme4 model candidates. We note that manually performing backwards selection in lme4 yielded the same model conclusions as those automated in glmulti, supporting the latter's utility for swift and reliable output. To refine further, we assessed relative importance values for predictors where values ≥0.8 signaled cumulative weighted importance in determining a top model (Calcagno and de Mazancourt, 2010). Because there was an apparent preference towards microhabitats with internal structures, e.g. hide boxes, bark slabs, and hibernacula, we created a separate dataset for only microhabitats with internal components and repeated the above steps. For this subset, we removed covariates T_s_ (only accounted for external surfaces) and T_s_-T_a_ and added T_a_, T_s_ in (internal microhabitat substrate temperature), and T_s_ ex-in (the difference in microhabitat external surface versus internal substrate temperature).

We derived several metrics to understand body temperature and thermal physiology of *T. rufipunctatus* (Objective 2). We first tested for regional heterothermy of integuments with a two-way type III repeated-measures analysis of variance (rmANOVA) with Greenhouse-Geisser corrections for sphericity via the afex package (Singmann et al., 2024); we only included individuals that had head, trunk, and tail measurements. We included exposure (i.e. exposed versus hidden) as a grouping factor, which may help account for potential heating versus cooling behaviors. We used averaged T_b_ values derived from thermographs for each integument, which can better account for heterothermy (Barroso et al., 2016; Blais et al., 2023a; Rowe et al., 2020).

We estimated total mean T_b_ per individual by averaging integument temperatures; at least one of head or trunk integuments was always used. We performed single-sample Z tests to compare the sample population mean T_b_ in this study to estimates (μ, s.d.) obtained from wild *T. rufipunctatus* populations (Fleharty, 1967; Hibbitts et al., 2009; Rosen, 1991; USFWS, 2014). We considered preferred body temperature (T_set_) as the median T_b_ value and the T_set-range_ as the first and last quartiles, i.e. interquartile range (Blouin-Demers and Weatherhead, 2001; Hertz et al., 1993; Taylor et al., 2021). We estimated thermoregulatory accuracy 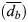 in two ways: first, we generated the absolute value difference between the average median value (i.e. T_set_) from mean T_b_ (Hertz et al., 1993; Taylor et al., 2021); we also derived the raw difference in T_set_-T_b_, which may better estimate poor thermoregulation at preferred warmer temperatures (d_b_ <0) or poor cooling to the preferred body temperature (d_b_ >0; Ivey et al., 2020; Taylor et al., 2021). We conservatively inferred VT_max_ (i.e. voluntary maximum temperature before animal retreats to shelter to avoid further heating; Taylor et al., 2021) by averaging the maximum T_b_ per survey date (Ivey et al., 2020); we acknowledge these estimates are relative and may not encompass the full suite of thermal physiology and confounding effects experienced in a further-controlled thermal arena or natural setting (Terblanche et al., 2011).

To understand environmental factors that may influence T_b_ in *T. rufipunctatus* (Objective 3), we followed Blais et al. (2023a) by conducting a candidate suite of general linear mixed models (LMM) with Gaussian distribution. Predictors included occupied (surface) perch temperature (T_perch_), T_s_-T_a_, rH, P_b_, exposed, shared, and month; we set surveys per shift (morning versus afternoon) as random effects. Because T_w_ was correlated with both T_perch_ and T_a_, and gartersnakes were seldom observed in water, we did not include it as a model parameter. We omitted standalone T_a_ as a predictor due to high correlation with, and lower performance (e.g. AICc, *R*^2^) than T_perch_; perch temperature has shown to be a stronger predictor of T_b_ than T_a_ for other regional herpetofauna (Blais et al., 2023a). We again used glmulti, AICc, and predictor relative importance as described above to select an optimal model among candidates. We used sjPlot (Lüdecke, 2024) to predict and plot values from resulting models.

To examine factors that may influence exposed (surface-visible) behavior – which may help guide field surveillance – we assessed logistic GLMM candidates in glmulti with the following environmental predictors: P_b_, rH, T_a_, T_s_ ex-in, and shift; we set month and survey as random effects. Finally, for aggregations (*n* ≥2, Gregory, 2004) in *T. rufipunctatus* (Objective 4), we conducted a series of GLMMs in glmulti in two parts. First, we asked what influences aggregations at microhabitats (binary: solitary versus aggregations ≥2)? Next, we asked what influences aggregation quantity, where the response variables were counts of individuals present at a given microhabitat? We used logistic GLMM for the former and Poisson GLMM for the latter. In both cases, predictors included T_perch_, T_s_-T_a_, rH, shift, month, and the average mean T_b_ from all individuals present; the latter aims to explain intrinsic influences. We set individual microhabitat ID and surveys as random effects. We repeated the model assessment workflow as described above. Data are presented as the mean±s.d. unless stated otherwise. We report Nakagawa & Schielzeth's *R*^2^ for optimal models. We performed all analyses in program R (R Core Team, 2021).

## Supplementary Material



10.1242/biolopen.062264_sup1 Supplementary information

## References

[BIO062264C1] Aleksiuk, M. (1977). Cold-induced aggregative behavior in the red-sided garter snake (*Thamnophis sirtalis parietalis*). *Herpetologica* 33, 98-101. https://www.jstor.org/stable/3891638

[BIO062264C2] Allard, R. A. and Wells, S. A. (2018). The Phoenix Zoo Story: building a legacy of conservation. In *The Ark and Beyond: The Evolution of Zoo and Aquarium Conservation* (ed. B. A. Minteer, J. Maienschein and J. P. Collins), pp. 169-177. Chicago: University of Chicago Press.

[BIO062264C3] Alujević, K., Garcia-Costoya, G., Ratia, N., Schmitz, E., Godkin, R. S., Gopal, A. C., Bujan, J. and Logan, M. L. (2025). Using aerial thermography to map terrestrial thermal environments in unprecedented detail. *Methods Ecol. Evol.* 16, 1688-1702. 10.1111/2041-210X.70096

[BIO062264C4] Archer, S. R. and Predick, K. I. (2008). Climate change and ecosystems of the Southwestern United States. *Rangelands* 30, 23-28. 10.2111/1551-501X(2008)30[23:CCAEOT]2.0.CO;2

[BIO062264C5] Aubret, F. and Shine, R. (2009). Causes and consequences of aggregation by neonatal tiger snakes (*Notechis scutatus*, Elapidae). *Austral. Ecol.* 34, 210-217. 10.1111/j.1442-9993.2008.01923.x

[BIO062264C6] Barroso, F. M., Carretero, M. A., Silva, F. and Sannolo, M. (2016). Assessing the reliability of thermography to infer internal body temperatures of lizards. *J. Therm. Biol.* 62, 90-96. 10.1016/j.jtherbio.2016.10.004 27839556

[BIO062264C7] Barroso, F. M., Riaño, G., Sannolo, M., Carretero, M. A. and Rato, C. (2020). Evidence from *Tarentola mauritanica* (Gekkota: Phyllodactylidae) helps validate thermography as a tool to infer internal body temperatures of lizards. *J. Therm. Biol.* 93, 102700. 10.1016/j.jtherbio.2020.102700 33077121

[BIO062264C8] Bates, D., Mächler, M., Bolker, B. M. and Walker, S. C. (2015). Fitting linear mixed-effects models using lme4. *J. Stat. Softw.* 67, 1-48. 10.18637/jss.v067.i01

[BIO062264C9] Bellard, C., Bertelsmeier, C., Leadley, P., Thuiller, W. and Courchamp, F. (2012). Impacts of climate change on the future of biodiversity. *Ecol. Lett.* 15, 365-377. 10.1111/j.1461-0248.2011.01736.x 22257223 PMC3880584

[BIO062264C10] Bennett, J. M., Sunday, J., Calosi, P., Villalobos, F., Martínez, B., Molina-Venegas, R., Araújo, M. B., Algar, A. C., Clusella-Trullas, S., Hawkins, B. A. et al. (2021). The evolution of critical thermal limits of life on Earth. *Nat. Commun.* 12, 1198. 10.1038/s41467-021-21263-8 33608528 PMC7895938

[BIO062264C114] Bickford, D., Howard, S. D., Ng, D. J. J. and Sheridan, J. A. (2010). Impacts of climate change on the amphibians and reptiles of Southeast Asia. *Biodivers. Conserv.* 19, 1043-1062. 10.1007/s10531-010-9782-4

[BIO062264C11] Blais, B. R. and Lashway, S. (2018). *Thamnophis rufipunctatus* (Narrow-headed Gartersnake) Shared Refuge. *Herpetol. Rev.* 49, 357-358.

[BIO062264C12] Blais, B. R. and Koprowski, J. L. (2024). Modeling a hot, dry future: Substantial range reductions in suitable environment projected under climate change for a semiarid riparian predator guild. *PLoS ONE* 19, e0302981. 10.1371/journal.pone.0302981 38709740 PMC11073737

[BIO062264C13] Blais, B. R., Wells, S. A., Poynter, B. M., Koprowski, J. L., Garner, M. M. and Allard, R. A. (2022). Adaptive management in a conservation breeding program: Mimicking habitat complexities facilitates reproductive success in narrow-headed gartersnakes (*Thamnophis rufipunctatus*). *Zoo Biol.* 41, 346-353. 10.1002/zoo.21682 35037290

[BIO062264C14] Blais, B. R., Velasco, D. E., Frackiewicz, M. E., Low, A. Q. and Koprowski, J. L. (2023a). Assessing thermal ecology of herpetofauna across a heterogeneous microhabitat mosaic in a changing aridland riparian system. *Environ. Res. Ecol.* 2, 035001. 10.1088/2752-664X/ace6a3

[BIO062264C15] Blais, B. R., Wells, S. A., Poynter, B. M., Harris, T. R., Allard, R. A. and Koprowski, J. L. (2023b). Bridging conservation across the ex situ–in situ spectrum: Insights into the reproductive ecology of the threatened narrow−headed gartersnake (*Thamnophis rufipunctatus*). *Zoo Biol.* 42, 429-439. 10.1002/zoo.21747 36536594

[BIO062264C16] Blais, B. R., Bubac, C. M., Wells, S. A., Johnson, A. C., Morandini, M. and Koprowski, J. L. (2025). Reach and effectiveness of conservation translocations when founder animals are sourced from zoos. *Anim. Conserv*. 10.1111/acv.70011

[BIO062264C17] Blouin-Demers, G. and Weatherhead, P. J. (2001). Thermal ecology of black rat snakes (*Elaphe obsoleta*) in a thermally challenging environment. *Ecology* 82, 3025-3043. 10.1890/0012-9658(2001)082[3025:TEOBRS]2.0.CO;2

[BIO062264C18] Bodensteiner, B. L., Agudelo-Cantero, G. A., Andis Arrietta, A. Z., Gunderson, A. R., Muñoz, M. M., Refsnider, J. M. and Gangloff, E. J. (2021). Thermal adaptation revisited: how conserved are thermal traits of reptiles and amphibians? *J. Exp. Zool. Part A Ecol. Integr. Physiol.* 335, 173-194. 10.1002/jez.2414 32970931

[BIO062264C19] Bogert, C. M. (1959). How reptiles regulate their body temperature. *Sci. Am.* 200, 105-120. 10.1038/scientificamerican0459-105

[BIO062264C20] Burghardt, G. M. (2013). Environmental enrichment and cognitive complexity in reptiles and amphibians: Concepts, review, and implications for captive populations. *Appl. Anim. Behav. Sci.* 147, 286-298. 10.1016/j.applanim.2013.04.013

[BIO062264C21] Calcagno, V. (2020). glmulti: Model Selection and Multimodel Inference Made Easy. Available at: https://cran.r-project.org/package=glmulti.

[BIO062264C22] Calcagno, V. and de Mazancourt, C. (2010). glmulti: an R Package for easy automated model selection with (Generalized) linear models. *J. Stat. Softw.* 34, 1-29. 10.18637/jss.v034.i12

[BIO062264C23] Camacho, A., Rusch, T., Ray, G., Telemeco, R. S., Trefaut, M. and Angilletta, M. J. (2018). Measuring behavioral thermal tolerance to address hot topics in ecology, evolution, and conservation. *J. Therm. Biol.* 73, 71-79. 10.1016/j.jtherbio.2018.01.009 29549993

[BIO062264C24] Campobello, D., Lindström, J., Di Maggio, R. and Sarà, M. (2017). An integrated analysis of micro- and macrohabitat features as a tool to detect weatherdriven constraints: a case study with cavity nesters. *PLoS ONE* 12, e0174090. 10.1371/journal.pone.0174090 28319183 PMC5358771

[BIO062264C25] Chiszar, D., Smith, H. M. and Radcliffe, C. W. (1993). Zoo and laboratory experiments on the behavior of snakes: Assessments of competence in captive-raised animals. *Integr. Comp. Biol.* 33, 109-116. 10.1093/icb/33.2.109

[BIO062264C26] Choquette, J. D., Litzgus, J. D., Gui, J. X. Y. and Pitcher, T. E. (2023). A systematic review of snake translocations to identify potential tactics for reducing postrelease effects. *Conserv. Biol.* 37, e14016. 10.1111/cobi.14016 36436192 PMC10100070

[BIO062264C27] Christian, K. A., Tracy, C. R. and Tracy, C. R. (2016). Body temperatures and the thermal environment. In *Reptile Ecology and Conservation: A Handbook of Techniques* (ed. C. K. Dodd Jr), pp. 337-351. Oxford: Oxford University Press.

[BIO062264C28] Cook, B. I., Ault, T. R. and Smerdon, J. E. (2015). Unprecedented 21st century drought risk in the American Southwest and Central Plains. *Sci. Adv.* 1, e1400082. 10.1126/sciadv.1400082 26601131 PMC4644081

[BIO062264C29] Costanzo, J. P. (1989). Effects of humidity, temperature, and submergence behavior on survivorship and energy use in hibernating garter snakes, *Thamnophis sirtalis*. *Can. J. Zool.* 67, 2486-2492. 10.1139/z89-351

[BIO062264C30] Cowles, R. B. and Bogert, C. M. (1944). A preliminary study of the thermal requirements of desert reptiles. *Bull. Am. Museum Nat. Hist.* 83, 261-296. 10.1086/394795

[BIO062264C31] Cox, C. L., Chung, A. K., Davoll, M. E., Dehart, S. A., Gerardi, S. T., Ly, T. K., Moxley, K., Nipper, P. T., Novak, D. R., Reeves, P. F. et al. (2023). A diminutive snake species can maintain regional heterothermy in both homogeneous and heterogeneous thermal environments. *J. Exp. Biol.* 226, jeb245380. 10.1242/jeb.245380 37249067

[BIO062264C32] Cox, C. L., Chung, A. K., Bindrim, A., Davidson, G. G., Dean, S. M., Haines, K. C., Heise, A., Mauer, E., Pfennig, K. S., Sorrell, E. E. et al. (2024). Temperature dependence of regional heterothermy in a diminutive ectotherm. *J. Exp. Biol.* 227, jeb247759. 10.1242/jeb.247759 39324342

[BIO062264C33] Cox, N., Young, B. E., Bowles, P., Fernandez, M., Marin, J., Rapacciuolo, G., Böhm, M., Brooks, T. M., Hedges, S. B., Hilton-Taylor, C. et al. (2022). A global reptile assessment highlights shared conservation needs of tetrapods. *Nature* 605, 285-290. 10.1038/s41586-022-04664-7 35477765 PMC9095493

[BIO062264C115] Davis, M. A., Douglas, M. R., Webb, C. T., Collyer, M. L., Holycross, A. T., Painter, C. W., Kamees, L. K. and Douglas, M. E. (2015). Nowhere to go but up: impacts of climate change on demographics of a short-range endemic (*Crotalus willardi obscurus*) in the sky-islands of southwestern North America. *PLoS One* 10, e0131067. 10.1371/journal.pone.0131067 26114622 PMC4482755

[BIO062264C34] Davis, J. R., Taylor, E. N. and DeNardo, D. F. (2008). An automated temperature-based option for estimating surface activity and refuge use patterns in free-ranging animals. *J. Arid Environ.* 72, 1414-1422. 10.1016/j.jaridenv.2008.02.018

[BIO062264C35] Dubiner, S., Aguilar, R., Anderson, R. O., Arenas Moreno, D. M., Avila, L. J., Boada-Viteri, E., Castillo, M., Chapple, D. G., Chukwuka, C. O., Cree, A. et al. (2024). A global analysis of field body temperatures of active squamates in relation to climate and behaviour. *Glob. Ecol. Biogeogr.* 33, e13808. 10.1111/geb.13808

[BIO062264C36] Eisenberg, J. F. and Kleiman, D. G. (1977). The usefulness of behaviour studies in developing captive breeding programmes for mammals. *Int. Zoo Yearb.* 17, 81-89. 10.1111/j.1748-1090.1977.tb00871.x

[BIO062264C37] Fleharty, E. D. (1967). Comparative ecology of *Thamnophis elegans*, *T. cyrtopsis*, and *T. rufipunctatus* in New Mexico. *Southwest. Nat.* 12, 207-229. 10.2307/3669111

[BIO062264C38] Gardner, M. G., Pearson, S. K., Johnston, G. R. and Schwarz, M. P. (2016). Group living in squamate reptiles: a review of evidence for stable aggregations. *Biol. Rev.* 91, 925-936. 10.1111/brv.12201 26052742

[BIO062264C39] Georgescu, M., Broadbent, A. M. and Balling, R. C. Jr (2022). Effect of increased greenhouse gas concentration on mean, extreme, and timing of precipitation over Arizona (USA). *Int. J. Climatol.* 42, 3776-3792. 10.1002/joc.7444

[BIO062264C40] Goldberg, S. R. (2003). *Thamnophis rufipunctatus* (narrow-headed garter snake). Reproduction. *Herpetol. Rev.* 34, 158.

[BIO062264C41] Goller, M., Goller, F. and French, S. S. (2014). A heterogeneous thermal environment enables remarkable behavioral thermoregulation in *Uta stansburiana*. *Ecol. Evol.* 4, 3319-3329. 10.1002/ece3.1141 25535549 PMC4228607

[BIO062264C42] Goulet, C. T., Thompson, M. B., Michelangeli, M., Wong, B. B. M. and Chapple, D. G. (2017). Thermal physiology: A new dimension of the pace-of-life syndrome. *J. Anim. Ecol.* 86, 1269-1280. 10.1111/1365-2656.12718 28626934

[BIO062264C43] Graves, B. M. and Duvall, D. (1995). Aggregation of squamate reptiles associated with gestation, oviposition, and parturition. *Herpetol. Monogr.* 9, 102-119. 10.2307/1466999

[BIO062264C44] Gregory, P. T. (1990). Temperature differences between head and body in garter snakes (*Thamnophis*) at a den in central British Columbia. *J. Herpetol.* 24, 241-245. 10.2307/1564389

[BIO062264C45] Gregory, P. T. (2004). Analysis of patterns of aggregation under cover objects in an assemblage of six species of snakes. *Herpetologica* 60, 178-186. 10.1655/02-101

[BIO062264C46] Gregory, P. T. (2016). Responses of natricine snakes to predatory threat: A mini-review and research prospectus. *J. Herpetol.* 50, 183-195. 10.1670/15-103

[BIO062264C47] Griffis-Kyle, K. L., Mougey, K., Vanlandeghem, M., Swain, S. and Drake, J. C. (2018). Comparison of climate vulnerability among desert herpetofauna. *Biol. Conserv.* 225, 164-175. 10.1016/j.biocon.2018.06.009

[BIO062264C48] Gross, I. P., Wilson, A. E. and Wolak, M. E. (2024). The fitness consequences of wildlife conservation translocations a meta–analysis. *Biol. Rev.* 99, 348-371. 10.1111/brv.13025 37844577

[BIO062264C49] Harvey, D. S. and Weatherhead, P. J. (2010). Habitat selection as the mechanism for thermoregulation in a northern population of massasauga rattlesnakes (*Sistrurus catenatus*). *Ecoscience* 17, 411-419. 10.2980/17-4-3363

[BIO062264C50] Heller, S. B. and Halpern, M. (1982). Laboratory observations of aggregative behavior of garter snakes, *Thamnophis sirtalis*. *J. Comp. Physiol. Psychol.* 96, 967-983. 10.1037/0735-7036.96.6.967 7153391

[BIO062264C51] Hertz, P. E., Huey, R. B. and Stevenson, R. D. (1993). Evaluating temperature regulation by field-active ectotherms: the fallacy of the inappropriate question. *Am. Nat.* 142, 796-818. 10.1086/285573 19425957

[BIO062264C52] Hibbitts, T. J., Painter, C. W. and Holycross, A. T. (2009). Ecology of a population of the narrow-headed garter snake (*Thamnophis rufipunctatus*) in New Mexico: catastrophic decline of a river specialist. *Southwest. Nat.* 54, 461-467. 10.1894/GC-195.1

[BIO062264C53] Hill, R. W., Wyse, G. A. and Anderson, M. (2016). *Animal Physiology*, 4th edn Sunderland: Sinauer Associates.

[BIO062264C54] Hof, C., Araújo, M. B., Jetz, W. and Rahbek, C. (2011). Additive threats from pathogens, climate and land-use change for global amphibian diversity. *Nature* 480, 516-519. 10.1038/nature10650 22089134

[BIO062264C55] Holycross, A. T., Nowak, E. M., Christman, B. L. and Jennings, R. D. (2020). *Thamnophis rufipunctatus*: Mogollon narrow-headed gartersnake. In *Snakes of Arizona* (ed. A. T. Holycross and J. C. Mitchell), pp. 440-455. Rodeo: ECO Publishing.

[BIO062264C56] Huey, R. B., Peterson, C. R., Arnold, S. J. and Porter, W. P. (1989). Hot rocks and not-so-hot rocks: Retreat-site selection by garter snakes and its thermal consequences. *Ecology* 70, 931-944. 10.2307/1941360

[BIO062264C57] Ivey, K. N., Cornwall, M., Crowell, H., Ghazian, N., Nix, E., Owen, M., Zuliani, M., Lortie, C. J., Westphal, M. and Taylor, E. (2020). Thermal ecology of the federally endangered blunt-nosed leopard lizard (*Gambelia sila*). *Conserv. Physiol.* 8, coaa014. 10.1093/conphys/coaa014 33649711 PMC7047230

[BIO062264C58] Kearney, M., Shine, R. and Porter, W. P. (2009). The potential for behavioral thermoregulation to buffer “cold-blooded” animals against climate warming. *Proc. Natl. Acad. Sci. USA* 106, 3835-3840. 10.1073/pnas.0808913106 19234117 PMC2656166

[BIO062264C59] Keppel, G., Mokany, K., Wardell-Johnson, G. W., Phillips, B. L., Welbergen, J. A. and Reside, A. E. (2015). The capacity of refugia for conservation planning under climate change. *Front. Ecol. Environ.* 13, 106-112. 10.1890/140055

[BIO062264C60] Krochmal, A. R. and Bakken, G. S. (2003). Thermoregulation is the pits: Use of thermal radiation for retreat site selection by rattlesnakes. *J. Exp. Biol.* 206, 2539-2545. 10.1242/jeb.00471 12819261

[BIO062264C61] Lelièvre, H., Blouin-Demers, G., Bonnet, X. and Lourdais, O. (2010). Thermal benefits of artificial shelters in snakes: A radiotelemetric study of two sympatric colubrids. *J. Therm. Biol.* 35, 324-331. 10.1016/j.jtherbio.2010.06.011

[BIO062264C62] Lüdecke, D. (2024). sjPlot: Data Visualization for Statistics in Social Science. Available at: https://cran.r-project.org/package=sjPlot.

[BIO062264C63] Luna, S. and Font, E. (2013). Use of an infrared thermographic camera to measure field body temperatures of small lacertid lizards. *Herpetol. Rev.* 44, 59-62.

[BIO062264C64] Mazzamuto, M. V., Morandini, M., Lampman, W., Wauters, L. A., Preatoni, D., Koprowski, J. L. and Martinoli, A. (2023). Use of infrared thermography to detect reactions to stressful events: does animal personality matter? *Integr. Zool.* 19, 224-239. 10.1111/1749-4877.12735 37248795

[BIO062264C65] McCafferty, D. J., Koprowski, R., Herborn, K., Tattersall, G. J., Jerem, P. and Nord, A. (2021). Editorial: Advances in thermal imaging. *J. Therm. Biol.* 102, 103109. 10.1016/j.jtherbio.2021.103109 34863474

[BIO062264C66] Minteer, B. A., Maienschein, J. and Collins, J. P. (eds) (2018). *The Ark and Beyond: The Evolution of Zoo and Aquarium Conservation*. Chicago: The University of Chicago Press.

[BIO062264C67] Mitchell, W. F. and Clarke, R. H. (2019). Using infrared thermography to detect night-roosting birds. *J. F. Ornithol.* 90, 39-51. 10.1111/jofo.12285

[BIO062264C68] Mitchell, D., Maloney, S. K., Snelling, E. P., Hetem, R. S., Strauss, W. M. and Fuller, A. (2018). Revisiting concepts of thermal physiology: Predicting responses of mammals to climate change. *J. Anim. Ecol.* 87, 956-973. 10.1111/1365-2656.12818 29479693

[BIO062264C116] Mitchell, N. J., Rodriguez, N., Kuchling, G., Arnall, S. G. and Kearney, M. R. (2016). Reptile embryos and climate change: modelling limits of viability to inform translocation decisions. *Biol. Conserv.* 204, 134-147. 10.1016/j.biocon.2016.04.004

[BIO062264C69] Mochales-Riaño, G., Barroso, F. M., Marques, V., Telea, A. E., Sannolo, M., Rato, C. and Carretero, M. A. (2024). Novel method to investigate thermal exchange rates in small, terrestrial ectotherms: A proof-of-concept on the gecko *Tarentola mauritanica*. *PLoS ONE* 19, e0316283. 10.1371/journal.pone.0316283 39724253 PMC11670986

[BIO062264C70] Monge, O., Caro, S. P. and Charmantier, A. (2025). What does infrared thermography tell us about the evolutionary potential of heat tolerance in endotherms? *Evol. Lett.* 9, 184-188. 10.1093/evlett/qrae070 40191413 PMC11968186

[BIO062264C71] Murphy, J., Adler, K. and Collins, J. T. (eds). (1994). *Captive Management Conservation of Amphibians and Reptiles*. Ithaca: Society for the Study of Amphibians & Reptiles.

[BIO062264C72] Mushinsky, H. R. and McCoy, E. D. (2016). Measuring microhabitats used by non-avian reptiles. In *Reptile Ecology and Conservation: A Handbook of Techniques* (ed. C. K. Dodd Jr), pp. 254-271. Oxford: Oxford University Press.

[BIO062264C73] Ortega, Z., Mencía, A., Martins, K., Soares, P., Ferreira, V. L. and Oliveira-Santos, L. G. (2019). Disentangling the role of heat sources on microhabitat selection of two Neotropical lizard species. *J. Trop. Ecol.* 35, 149-156. 10.1017/S0266467419000099

[BIO062264C74] Pacifici, M., Foden, W. B., Visconti, P., Watson, J. E. M., Butchart, S. H. M., Kovacs, K. M., Scheffers, B. R., Hole, D. G., Martin, T. G., Akcakaya, H. R. et al. (2015). Assessing species vulnerability to climate change. *Nat. Clim. Chang.* 5, 215-225. 10.1038/NCLIMATE2448

[BIO062264C75] Péchy, T., Halpern, B., Sós, E. and Walzer, C. (2015). Conservation of the Hungarian meadow viper *Vipera ursinii rakosiensis*. *Int. Zoo Yearb.* 49, 89-103. 10.1111/izy.12088

[BIO062264C76] Pecl, G., Araujo, M. B., Bell, J. D., Blanchard, J., Bonebrake, T. C., Chen, I.-C., Clark, T. D., Colwell, R. K., Danielsen, F., Evengård, B. et al. (2017). Biodiversity redistribution under climate change: Impacts on ecosystems and human well-being. *Science* 355, eaai9214. 10.1126/science.aai9214 28360268

[BIO062264C77] Pilliod, D. S., Jeffries, M. I., Arkle, R. S. and Olson, D. H. (2024). Climate futures for lizards and snakes in western North America may result in new species management issues. *Ecol. Evol.* 14, e70379. 10.1002/ece3.70379 39403261 PMC11471426

[BIO062264C78] Playà-Montmany, N. and Tattersall, G. J. (2021). Spot size distance and emissivity errors in field applications of infrared thermography. *Methods Ecol. Evol.* 12, 828-840. 10.1111/2041-210X.13563

[BIO062264C79] Pritchard, D. J., Fa, J. E., Oldfield, S. and Harrop, S. R. (2011). Bring the captive closer to the wild: redefining the role of ex situ conservation. *Oryx* 46, 18-23. 10.1017/S0030605310001766

[BIO062264C80] R Core Team (2021). R: A language and environment for statistical computing. R Foundation for Statistical Computing. Available at: https://www.r-project.org/.

[BIO062264C81] Rabier, R., Erlichman, A., Lesobre, L. and Robert, A. (2022). The necessity of considering founder kinships in conservation breeding programs. *Anim. Conserv.* 25, 759-770. 10.1111/acv.12779

[BIO062264C82] Radchuk, V., Reed, T., Teplitsky, C., van de Pol, M., Charmantier, A., Hassall, C., Adamík, P., Adriaensen, F., Ahola, M. P., Arcese, P. et al. (2019). Adaptive responses of animals to climate change are most likely insufficient. *Nat. Commun.* 10, 3109. 10.1038/s41467-019-10924-4 31337752 PMC6650445

[BIO062264C83] Reading, R. P., Miller, B. and Shepherdson, D. (2013). The value of enrichment to reintroduction success. *Zoo Biol.* 32, 332-341. 10.1002/zoo.21054 23426786

[BIO062264C84] Refsnider, J. M., Clifton, I. T. and Vazquez, T. K. (2019). Developmental plasticity of thermal ecology traits in reptiles: Trends, potential bene fits, and research needs. *J. Therm. Biol.* 84, 74-82. 10.1016/j.jtherbio.2019.06.005 31466792

[BIO062264C85] Reiserer, R. S., Schuett, G. W. and Earley, R. L. (2008). Dynamic aggregations of newborn sibling rattlesnakes exhibit stable thermoregulatory properties. *J. Zool.* 274, 277-283. 10.1111/j.1469-7998.2007.00383.x

[BIO062264C86] Roe, J. H., Frank, M. R. and Kingsbury, B. A. (2015). Experimental evaluation of captive-rearing practices to improve success of snake reintroductions. *Herpetol. Conserv. Biol.* 10, 711-722.

[BIO062264C87] Rosen, P. C. (1991). Field study of thermal preferenda in garter snakes (*Thamnophis*). *J. Herpetol.* 25, 301-312. 10.2307/1564588

[BIO062264C88] Rossman, D. A., Ford, N. B. and Seigel, R. A. (1996). *The Garter Snakes: Evolution and Ecology*. Norman: University of Oklahoma Press.

[BIO062264C89] Row, J. R. and Blouin-Demers, G. (2006). Thermal quality influences habitat selection at multiple spatial scales in milksnakes. *Ecoscience* 13, 443-450. 10.2980/1195-6860(2006)13[443:TQIHSA]2.0.CO;2

[BIO062264C90] Rowe, J. W., Clark, D. L., Martin, C. E. and Valle, C. (2020). Diel and seasonal variations in the thermal biology of San Cristobal Lava Lizards (*Microlophus bivittatus*). *J. Therm. Biol.* 88, 102518. 10.1016/j.jtherbio.2020.102518 32125995

[BIO062264C91] Ryan, M. J., Smith, A. B., Lashway, S., Smith, K. K., Riddle, S. B., Akins, C. M., Blais, B. R. and Krahn, K. T. (2019). *A five-year narrow-headed gartersnake (Thamnophis rufipunctatus) survey summary from Canyon Creek, Arizona*. Nongame and Endangered Wildlife Program Technical Report 323. Arizona Game and Fish Department, Phoenix, Arizona.

[BIO062264C92] Sannolo, M., Mangiacotti, M., Sacchi, R. and Scali, S. (2014). Keeping a cool mind: Head-body temperature differences in the common wall lizard. *J. Zool.* 293, 71-79. 10.1111/jzo.12121

[BIO062264C93] Sannolo, M., Ponti, R. and Carretero, M. A. (2019). Waitin’ on a sunny day: Factors affecting lizard body temperature while hiding from predators. *J. Therm. Biol.* 84, 146-153. 10.1016/j.jtherbio.2019.07.001 31466747

[BIO062264C94] Scheffers, B. R., Edwards, D. P., Diesmos, A., Williams, S. E. and Evans, T. A. (2014). Microhabitats reduce animal's exposure to climate extremes. *Glob. Chang. Biol.* 20, 495-503. 10.1111/gcb.12439 24132984

[BIO062264C95] Scheffers, B. R., Edwards, D. P., MacDonald, S. L., Senior, R. A., Andriamahohatra, L. R., Roslan, N., Rogers, A. M., Haugaasen, T., Wright, P. and Williams, S. E. (2016). Extreme thermal heterogeneity in structurally complex tropical rain forests. *Biotropica* 49, 35-44. 10.1111/btp.12355

[BIO062264C96] Scholander, P. F., Hock, R., Walters, V., Johnson, F. and Irving, L. (1950). Heat regulation in some arctic and tropical mammals and birds. *Biol. Bull.* 99, 237-258. 10.2307/1538741 14791422

[BIO062264C97] Signore, E., Clark, R. W. and Schraft, H. A. (2020). Temperature-based ambush site selection in sidewinder rattlesnakes (*Crotalus cerastes*). *Southwest. Nat.* 65, 282-287. 10.1894/0038-4909-65.3-4.282

[BIO062264C98] Singmann, H., Bolker, B., Westfall, J., Aust, F. and Ben-Shachar, M. S. (2024). afex: Analysis of Factorial Experiments. Available at: https://cran.r-project.org/package=afex.

[BIO062264C99] Skinner, M. and Miller, N. (2020). Aggregation and social interaction in garter snakes (*Thamnophis sirtalis sirtalis*). *Behav. Ecol. Sociobiol.* 74, 51. 10.1007/s00265-020-2827-0

[BIO062264C100] Spooner, S. L., Walker, S. L., Dowell, S. and Moss, A. (2023). The value of zoos for species and society: the need for a new model. *Biol. Conserv.* 279, 109925. 10.1016/j.biocon.2023.109925

[BIO062264C101] Suggitt, A. J., Wilson, R. J., Isaac, N. J. B., Beale, C. M., Auffret, A. G., August, T., Bennie, J. J., Crick, H. Q. P., Duffield, S., Fox, R. et al. (2018). Extinction risk from climate change is reduced by microclimatic buffering. *Nat. Clim. Chang.* 8, 713-717. 10.1038/s41558-018-0231-9

[BIO062264C102] Tattersall, G. J. (2016). Infrared thermography: A non-invasive window into thermal physiology. *Comp. Biochem. Physiol. Part A* 202, 78-98. 10.1016/j.cbpa.2016.02.022 26945597

[BIO062264C103] Tattersall, G. J., Cadena, V. and Skinner, M. C. (2006). Respiratory cooling and thermoregulatory coupling in reptiles. *Respir. Physiol. Neurobiol.* 154, 302-318. 10.1016/j.resp.2006.02.011 16574503

[BIO062264C104] Taylor, E. N., Viegas, L. M. D., Gangloff, E. J., Hall, J. M., Halpern, B., Massey, M. D., Rödder, D., Rollinson, N., Spears, S., Sun, B. et al. (2021). The thermal ecology and physiology of reptiles and amphibians: A user's guide. *J. Exp. Zool. Part A* 335, 13-44. 10.1002/jez.2396 32638552

[BIO062264C105] Terblanche, J. S., Hoffmann, A. A., Mitchell, K. A., Rako, L., Le Roux, P. C. and Chown, S. L. (2011). Ecologically relevant measures of tolerance to potentially lethal temperatures. *J. Exp. Biol.* 214, 3713-3725. 10.1242/jeb.061283 22031735

[BIO062264C106] USFWS (2014). Endangered and threatened wildlife and plants; threatened status for the northern mexican gartersnake and narrow-headed gartersnake; final rule. *Fed. Regist.* 79, 38678-38746. https://www.regulations.gov/document/FWS-R2-ES-2013-0071-0041

[BIO062264C107] USFWS (2021). Endangered and threatened wildlife and plants; designation of critical habitat for the narrow-headed gartersnake. *Fed. Regist.* 86, 58474-58523. https://www.regulations.gov/document/FWS-R2-ES-2020-0011-0056

[BIO062264C108] Venegas-Barrera, C. S., Sunny, A. and Manjarrez, J. (2025). Thermal ecology of the Mexican garter snake (*Thamnophis eques*): temporal and spatial variations. *PeerJ* 13, e18641. 10.7717/peerj.18641 39822974 PMC11737337

[BIO062264C109] Visser, M. E. (2008). Keeping up with a warming world; assessing the rate of adaptation to climate change. *Proc. R. Soc. B Biol. Sci.* 275, 649-659. 10.1098/rspb.2007.0997 PMC2409451 18211875

[BIO062264C110] Wood, D. A., Emmons, I. D., Nowak, E. M., Christman, B. L., Holycross, A. T., Jennings, R. D. and Vandergast, A. G. (2018). *Conservation genomics of the Mogollon Narrow-headed gartersnake (Thamnophis rufipunctatus) and Northern Mexican gartersnake (Thamnophis eques megalops)*. U.S. Geological Survey Open-File Report 2018 −1141. 10.3133/ofr20181141

[BIO062264C111] Wood, D. A., Christman, B. L., Jennings, R. D., Rose, J., Nowak, E., Schofer, J. and Vandergast, A. G. (2025a). Increased heterozygosity and body condition result from admixed translocation of the threatened Mogollon narrow-headed gartersnake (*Thamnophis rufipunctatus*). *Conserv. Genet.* 26, 403-418. 10.1007/s10592-025-01677-3

[BIO062264C112] Wood, D. A., Mitelberg, A. and Vandergast, A. G. (2025b). *Parentage and sibship relationships among captive snakes at the Phoenix Zoo—2024 Data Summary*. U.S. Geological Survey Data Report 1204. 10.3133/dr1204

[BIO062264C113] Woods, H. A., Dillon, M. E. and Pincebourde, S. (2015). The roles of microclimatic diversity and of behavior in mediating the responses of ectotherms to climate change. *J. Therm. Biol.* 54, 86-97. 10.1016/j.jtherbio.2014.10.002 26615730

