# Supplementary material for: Insights into the thermal ecology, physiology, and behavior of a threatened ectothermic specialist from a warming and drying ecoregion
Source: Biol Open. 2025 Dec 23;14(12):bio062264. doi: 10.1242/bio.062264 (PMC12772134; doi:10.1242/bio.062264)
Supplement: Supplementary information [file biolopen-14-062264-s1.pdf]

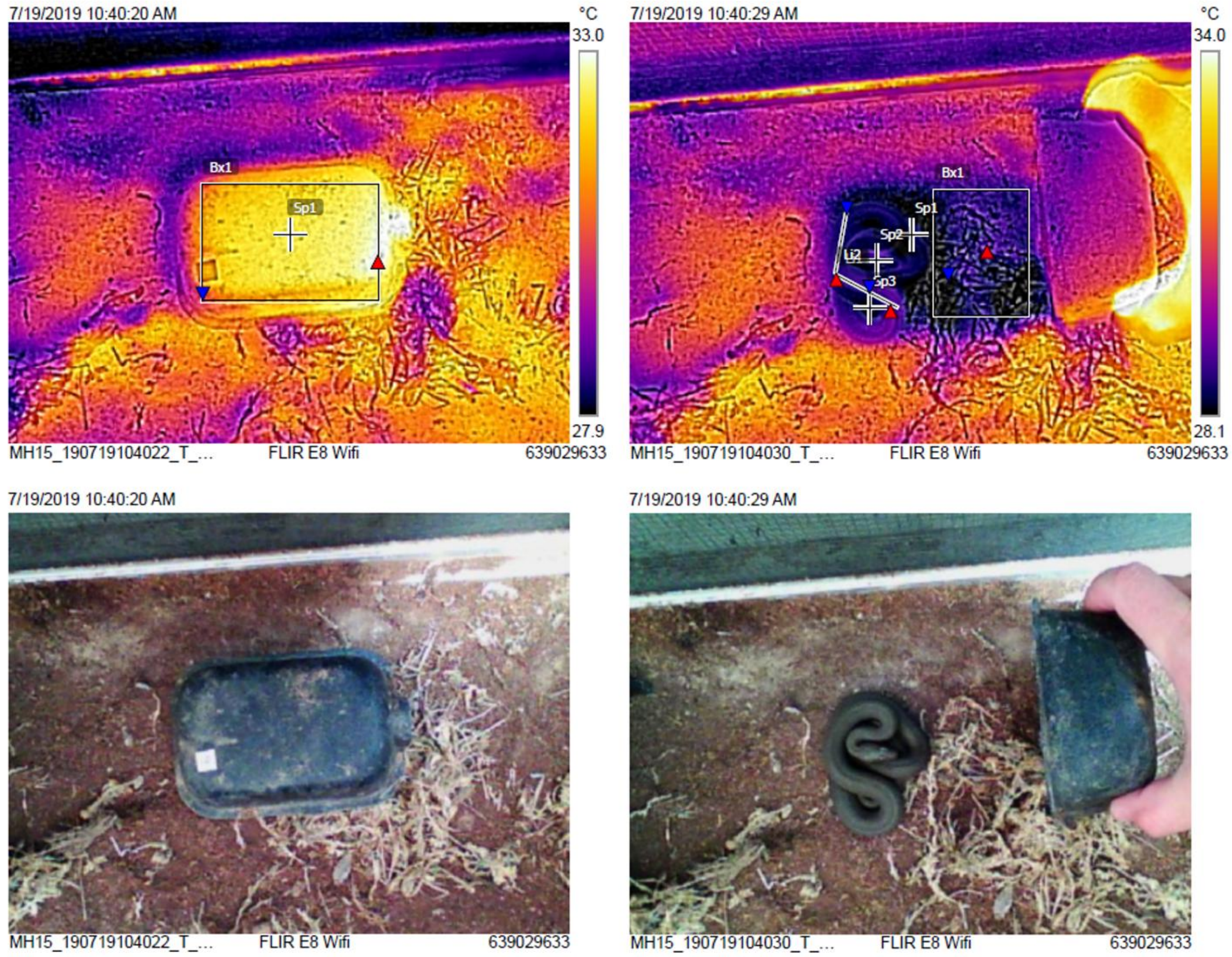

Fig. S1. Sample multispectral infrared thermography scans for *ex situ* colony of *Thamnophis rufipunctatus* at the Phoenix Zoo, 2019. Example multispectral thermogram/photograph pairings (above:below) from a FLIR E8 infrared thermal camera reflecting scans of a microhabitat external surface (left) before assessing internal substrate surface, occupied by a gartersnake (right). Spot, line, and polygon functions in FLIR TOOLS software are used to derive thermal values for microhabitat and individual, respectively.

Biology Open • Supplementary information

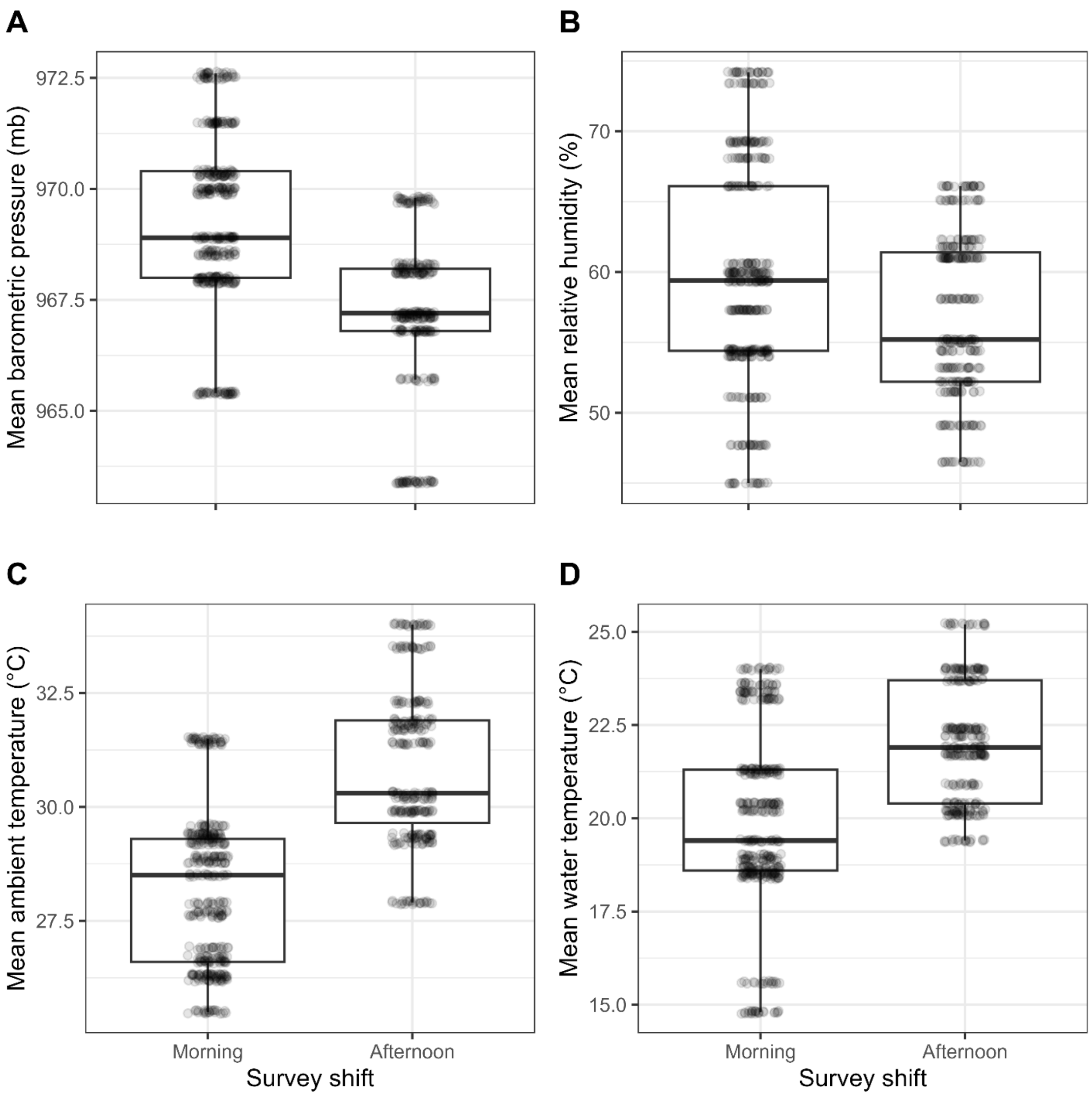

Fig. S2. Ambient enclosure conditions in *ex situ* mesocosms for *Thamnophis rufipunctatus* at the Phoenix Zoo, 2019. These data are partitioned by survey shift (morning vs. afternoon) for (A) barometric pressure (mb), (B) relative humidity (%), (C) ambient air temperature (°C), and (D) water surface temperature (°C). All ambient conditions were significantly different ($P < 0.05$) between shifts.

Biology Open • Supplementary information

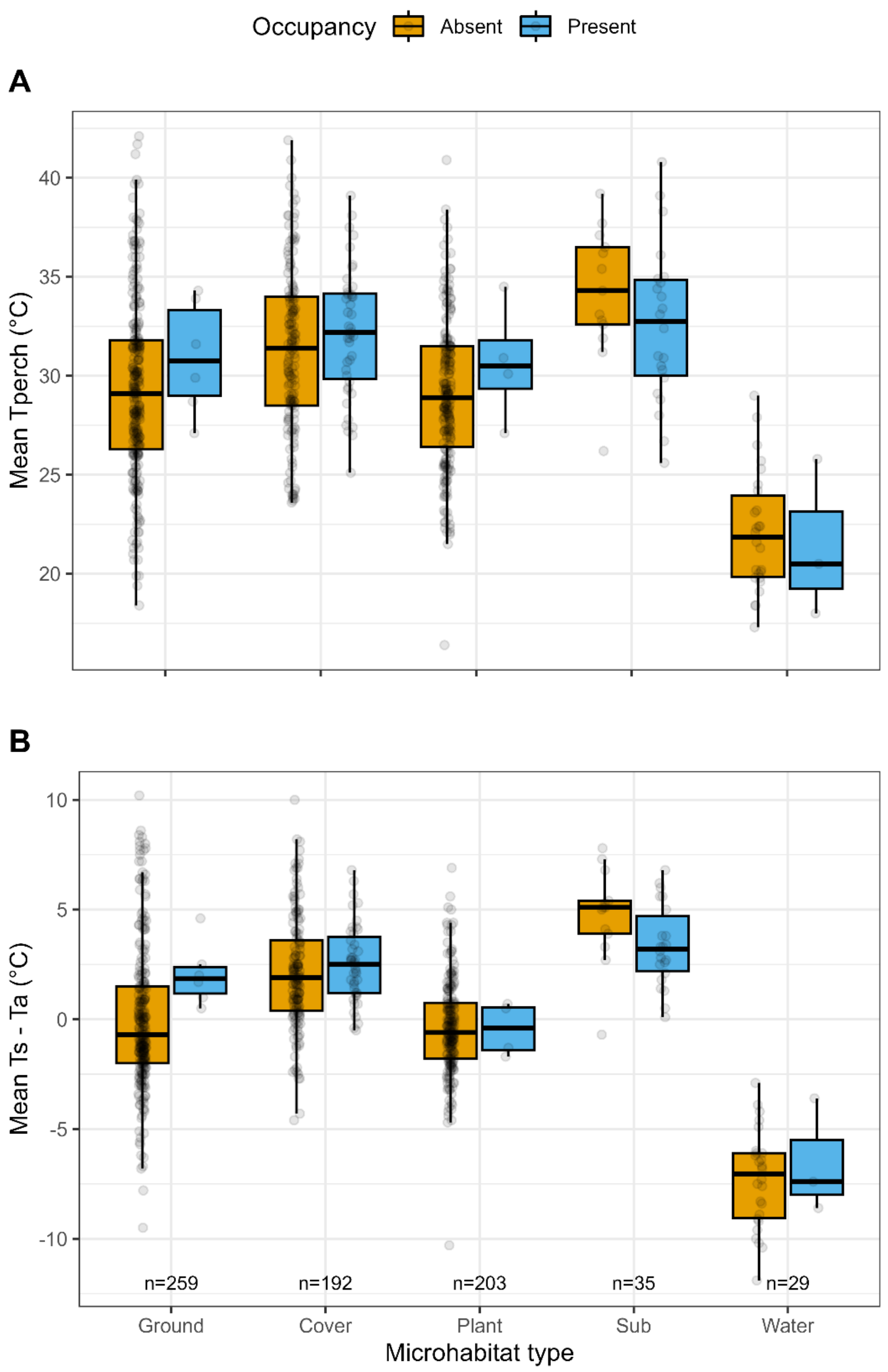

Fig. S3. Thermal relationships of microhabitats used by the *ex situ* colony of *Thamnophis rufipunctatus* at the Phoenix Zoo, 2019. Data relationships are partitioned by (A) microhabitat mean surface perch temperature (T_{perch}) and (B) the difference in surface to ambient air temperature (T_s-T_a). Colors depict occupied (blue) versus available (orange) microhabitats.

Biology Open • Supplementary information

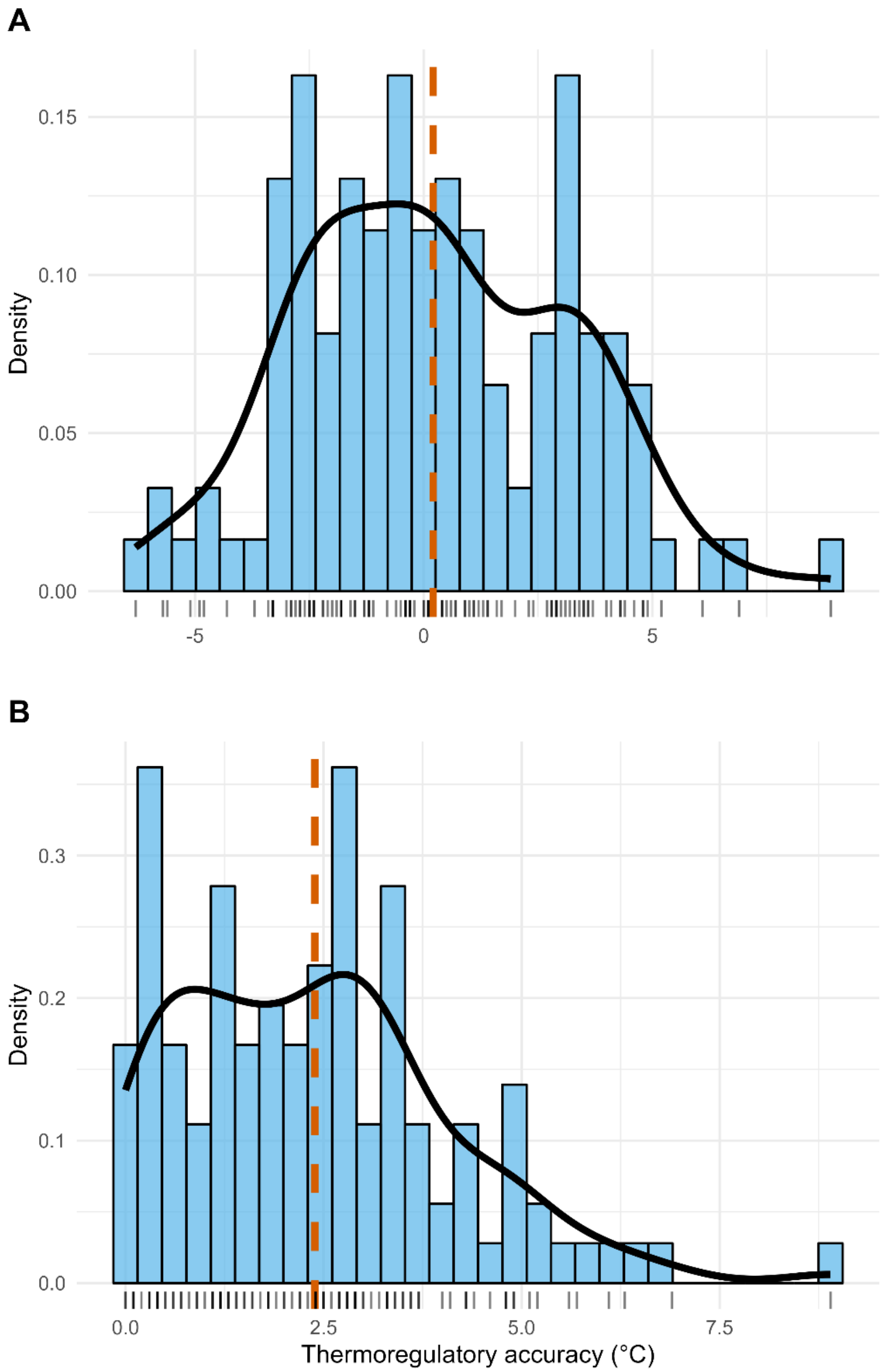

Fig. S4. Thermoregulatory accuracy (d_b) for *ex situ* colony of *Thamnophis rufipunctatus* at the Phoenix Zoo, 2019. Formulae: (A) $d_b = T_{set}$-T_b, (Ivey et al., 2020) and (B) $d_b = |T_{set} - T_b|$, (Hertz et al., 1993), where T_b is body temperature and T_{set} is the (sample population) average median body temperature (vertical lines). Zero values represent perfect thermoregulatory accuracy whereas departures reflect inefficiencies of warming (>0) or cooling (<0; Taylor et al. 2021). Tick marks along the x-axis represent individual datapoints.

Biology Open • Supplementary information

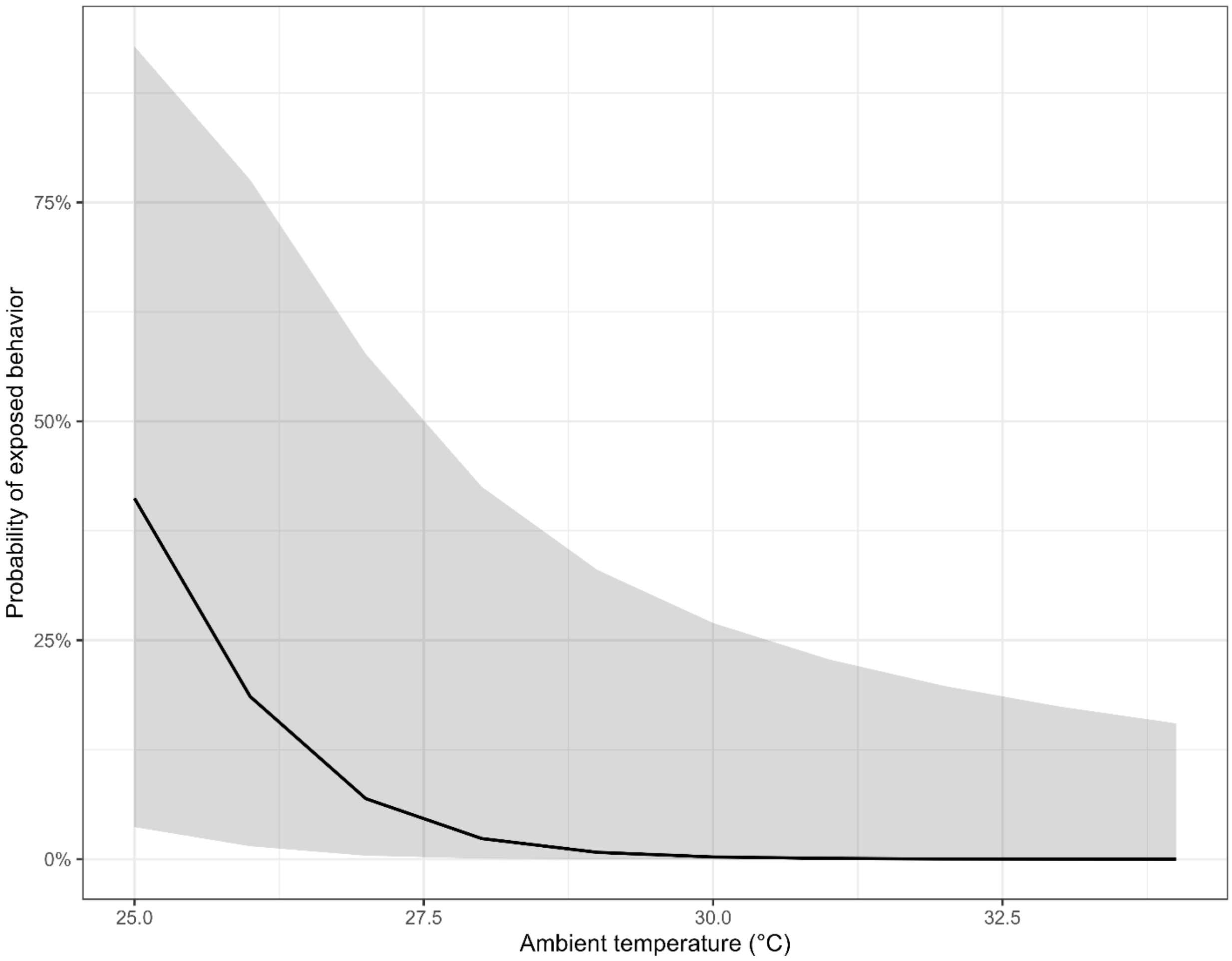

Fig. S5. Probability of exposed (i.e., surface-visible) behavior based on ambient air temperature for *ex situ* colony of *Thamnophis rufipunctatus* at the Phoenix Zoo, 2019. Probability of 100% reflects exposures whereas 0% indicates hidden individuals at a given microhabitat.

Table S1. Microhabitat surface temperature (°C) relationships in *ex situ* mesocosms for *Thamnophis rufipunctatus* at the Phoenix Zoo, 2019. Data represent sampling events (*n*), and mean (±s.d.) for mean external microhabitat surface temperature (T_s), difference in T_s to ambient air temperature (T_s - T_a), sampling events for microhabitats with internal structures (*n internal*; e.g., hide boxes), the mean ground surface temperature underlying microhabitats with internal structures (T_s *internal*), and the difference in microhabitat external versus internal surface temperature (T_s *ex-in*).

	Microhabitat Type				
	Cover	Ground	Plant	Sub	Water
n	192	259	203	35*	29
T_s	31.5 (4.0)	29.4 (4.5)	29.1 (3.8)	33.2* (3.8)	22.0 (3.1)
T_s - T_a	2.2 (2.5)	0.1 (3.3)	-0.4 (2.2)	3.9* (2.1)	-7.2 (2.2)
n (internal)	192	52	n/a	35	n/a
T_s (internal)	27.0 (3.8)	25.1 (3.5)	n/a	26.9 (3.5)	n/a
T_s (ex-in)	4.6 (1.8)	3.7 (1.4)	n/a	6.3 (2.0)	n/a

*external cover slab of subterranean hibernacula

Biology Open • Supplementary information

Table S2. Mixed effect logistic regression model tables for microhabitats occupied by *Thamnophis rufipunctatus* in *ex situ* microcosms at the Phoenix Zoo, 2019. The top 10 performing models are shown for full dataset of microhabitats occupied (Response variable = occu, above) and for a subset with only microhabitats that had an internal structure (Response variable = occu.int, below). Automated model selection was performed using corrected Akaike's Information Criterion (AICc) and relative model weight amongst a suite of global model candidates.

Response	Terms	AICc	weight
occu	type + (1|MH.ID)	312.5821	0.1876
	type + rH + (1|MH.ID)	313.0834	0.1460
	type + T_s-T_a + (1|MH.ID)	314.4819	0.0726
	type + shift + (1|MH.ID)	314.5104	0.0715
	type + T_s + (1|MH.ID)	314.6122	0.0680
	type + shift + rH + (1|MH.ID)	314.7867	0.0623
	type + T_s + rH + (1|MH.ID)	315.0657	0.0542
	type + T_s-T_a + rH + (1|MH.ID)	315.1048	0.0531
	type + T_s + T_s-T_a + (1|MH.ID)	315.7106	0.0393
	type + shift + T_s + rH + (1|MH.ID)	316.4064	0.0277
occu.int	type + (1|MH.ID)	198.6731	0.1065
	type + T_s in + T_a + (1|MH.ID)	199.3630	0.0754
	type + T_s in + T_s ex-in + T_a + (1|MH.ID)	199.9743	0.0556
	type + T_a + (1|MH.ID)	200.1097	0.0519
	type + rH + (1|MH.ID)	200.2512	0.0484
	type + shift + T_s in + T_a + (1|MH.ID)	200.3692	0.0456
	type + T_s in + (1|MH.ID)	200.6650	0.0393
	type + shift + (1|MH.ID)	200.6930	0.0388
	type + T_s ex-in + (1|MH.ID)	200.7255	0.0382
	type + shift + T_s in + T_s ex-in + T_a + (1|MH.ID)	201.1624	0.0307

Abbreviations: *occu* = occupied microhabitat (binary); *occu.int* = occupied microhabitat possessing internal structure (binary); *rH* = ambient relative humidity (±0.1%); *shift* = survey shift (morning/afternoon); T_a = ambient air temperature (±0.1°C); T_s = microhabitat (external) surface temperature (±0.1°C); T_s *ex-in* = difference in microhabitat external to internal surface temperature (±0.1°C); T_s *in* = microhabitat internal surface temperature (±0.1°C); T_s-T_a = difference in microhabitat external surface temperature to air temperature (±0.1°C); *type* = microhabitat type class (categorical). Unique microhabitats were modeled as random effects (1|MH.ID).

Biology Open • Supplementary information

Table S3. Mixed effect linear regression model tables explaining body temperature (response) of *Thamnophis rufipunctatus* in *ex situ* microcosms at the Phoenix Zoo, 2019. Automated model selection was performed using corrected Akaike's Information Criterion (AICc) and relative model weight amongst a suite of global candidate models.

Terms	AICc	weight
exposed + shared + T_{perch} + T_s-T_a + (1| shift/survey)	391.3218	0.2190
exposed + T_{perch} + T_s-T_a + (1| shift/survey)	392.5935	0.1159
exposed + shared + T_{perch} + T_s-T_a + P_b + (1| shift/survey)	392.9828	0.0954
exposed + shared + T_{perch} + T_s-T_a + rH + (1| shift/survey)	393.1614	0.0873
exposed + T_{perch} + T_s-T_a + P_b + (1| shift/survey)	394.0801	0.0551
exposed + shared + T_{perch} + T_s-T_a + month + (1| shift/survey)	394.0826	0.0551
exposed + shared + T_{perch} + T_s-T_a + rH +P_b + (1| shift/survey)	394.4052	0.0469
exposed + T_{perch} + T_s-T_a + rH + (1| shift/survey)	394.4766	0.0452
shared + T_{perch} + T_s-T_a + (1| shift/survey)	394.7276	0.0399
exposed + T_{perch} + T_s-T_a + month + (1| shift/survey)	395.2459	0.0308

Abbreviations: *exposed* = if individual was visibly exposed or hidden within a refuge (binary); *month* = month; P_b = barometric pressure (±0.1mb); *rH* = ambient relative humidity (±0.1%); *shared* = if individual was sharing a microhabitat with another (binary); T_{perch} = surface perch temperature (±0.1°C); T_s-T_a = difference in perch surface temperature to air temperature (±0.1°C). Unique shift (i.e., morning/afternoon) per survey were modeled as random effects (1|shift/survey).

Table S4. **Mixed effect linear regression model tables explaining exposed behavior (i.e., surface-visible; response) of *Thamnophis rufipunctatus* in *ex situ* microcosms at the Phoenix Zoo, 2019.** Automated model selection was performed using corrected Akaike's Information Criterion (AICc) and relative model weight amongst a suite of global candidate models.

Terms	AICc	weight
T_s ex-in + T_a + (1|month) + (1|survey)	46.8523	0.4606
shift + T_s ex-in + T_a + (1|month) + (1|survey)	49.0782	0.1514
T_s ex-in + T_a + rH + (1|month) + (1|survey)	49.0950	0.1501
T_s ex-in + (1|month) + (1|survey)	50.5348	0.0731
shift + T_s ex-in + (1|month) + (1|survey)	50.7664	0.0651
shift + T_s ex-in + T_a + rH+ (1|month) + (1|survey)	51.3639	0.0483
T_s ex-in + rH + (1|month) + (1|survey)	52.5472	0.0267
shift + T_s ex-in + rH + (1|month) + (1|survey)	52.6918	0.0248
T_a + (1|month) + (1|survey)	99.5753	<0.0001
T_a + rH + (1|month) + (1|survey)	100.8582	<0.0001

Abbreviations: P_b = barometric pressure (±0.1mb); *rH* = ambient relative humidity (±0.1%); *shift* = survey shift (morning/afternoon); T_a = ambient air temperature (±0.1°C); T_s *ex-in* = difference in microhabitat external to internal surface temperature (±0.1°C). Month and survey were modeled as random effects (1|month) + (1| survey).

Table S5. Mixed effect linear regression model tables explaining aggregation behavior of *Thamnophis rufipunctatus* in *ex situ* microcosms at the Phoenix Zoo, 2019. Models include instances of aggregation (binary, above) and aggregation counts (Poisson, below). Automated model selection was performed using corrected Akaike's Information Criterion (AICc) and relative model weight amongst a suite of global candidate models.

Response	Terms	AICc	weight
binary	T_b_avg + T_{perch} + (1|survey) + (1|MH.ID)	96.0750	0.1993
	T_b_avg + T_{perch} + rH + (1|survey) + (1|MH.ID)	97.5083	0.0973
	shift + T_b_avg + T_{perch} + (1|survey) + (1|MH.ID)	97.6881	0.0890
	T_b_avg + (1|survey) + (1|MH.ID)	97.8282	0.0829
	T_b_avg + T_{perch} + T_s-T_a + (1|survey) + (1|MH.ID)	98.4444	0.0609
	month + T_b_avg + T_{perch} + (1|survey) + (1|MH.ID)	99.2105	0.0416
	shift + T_b_avg + T_{perch} + rH + (1|survey) + (1|MH.ID)	99.6143	0.0340
	shift + T_b_avg + (1|survey) + (1|MH.ID)	99.6589	0.0332
	T_b_avg + T_{perch} + T_s-T_a + rH + (1|survey) + (1|MH.ID)	99.8860	0.0296
	T_b_avg + rH + (1|survey) + (1|MH.ID)	99.9085	0.0293
Poisson	T_{perch} + (1|survey) + (1|MH.ID)	211.0087	0.2269
	T_b_avg + T_{perch} + (1|survey) + (1|MH.ID)	212.4913	0.1081
	T_b_avg + (1|survey) + (1|MH.ID)	212.8669	0.0896
	shift + T_{perch} + (1|survey) + (1|MH.ID)	213.0706	0.0809
	T_{perch} + T_s-T_a + (1|survey) + (1|MH.ID)	213.2904	0.0725
	T_{perch} + rH + (1|survey) + (1|MH.ID)	213.2930	0.0724
	T_b_avg + T_{perch} + rH + (1|survey) + (1|MH.ID)	214.8006	0.0341
	T_b_avg + T_{perch} + T_s-T_a + (1|survey) + (1|MH.ID)	214.8384	0.0334
	shift + T_b_avg + T_{perch} + (1|survey) + (1|MH.ID)	214.8478	0.0333
	T_b_avg + T_s-T_a + (1|survey) + (1|MH.ID)	215.1006	0.0293

Abbreviations: *rH* = ambient relative humidity (±0.1%); *shift* = morning vs afternoon survey (binary); *Tb_avg* = average mean body temperature among individuals present; T_{perch} = surface perch temperature (±0.1°C); T_s-T_a = difference in perch surface temperature to air temperature (±0.1°C). Unique microhabitats and surveys were modeled as random effects (1|survey) + (1|MH.ID).

Biology Open • Supplementary information
